# Diagnostic value of exosome non-coding RNAs as non-invasive biomarkers in gastric cancer: a meta-analysis

**DOI:** 10.3389/fonc.2025.1570020

**Published:** 2025-06-12

**Authors:** Qiumiao Xu, Qinghong Yan, Zhijiang Dai, Xiaoting Chen, Tongwei Liu, Chengjin Peng, Guoxin Huang, Xinyao Liu, Jingbin Wang

**Affiliations:** ^1^ Department of Spleen and Stomach, Guangzhou University of Chinese Medicine Shenzhen Hospital (Futian), Shenzhen, China; ^2^ The Sixth Clinical Medical College, Guangzhou University of Chinese Medicine, Shenzhen, China

**Keywords:** exosomes, noncoding RNA, gastric cancer, biomarker, meta-analysis

## Abstract

**Purpose:**

Gastric cancer, characterised by a significant mortality rate, is one of the most prevalent malignant neoplasms globally. Exosomal non-coding RNAs play a key role in gastric carcinogenesis, metastasis and treatment resistance by regulating gene expression, remodelling the tumour microenvironment and mediating drug resistance. In the identification of early gastric cancer, these exosomal non-coding RNA molecules possess significant potential to be developed into biomarkers that do not entail invasive procedures.

**Methods:**

Our study undertook a comprehensive and profound literature examination in core databases like PubMed, Web of Science, ScienceDirect, Embase, Scopus, and Medline, with the aim of precisely evaluating the potential effectiveness of exosomal miRNAs, lncRNAs, and circRNAs in the diagnosis of gastric cancer.

**Results:**

A sum of 52 studies were incorporated, comprising 164 studies. These studies identified a total of 59 miRNAs, 17 lncRNAs, and 16 circRNAs. For miRNAs, sensitivity was estimated at 0.72 (95% CI, 0.69 - 0.76) and specificity at 0.80 (95% CI, 0.77 - 0.83). For lncRNAs, the sensitivity was 0.86 (95% CI, 0.84 - 0.87) and the specificity was 0.82 (95% CI, 0.80 - 0.83). In the case of circRNAs, the sensitivity and specificity were 0.71 (95% CI, 0.63 - 0.78) and 0.88 (95% CI, 0.81 - 0.93) respectively. The AUC for miRNAs was calculated as 0.83. As for lncRNAs, its AUC was established to be 0.89. Regarding circRNAs, the determined AUC was 0.86.

**Conclusion:**

These results confirm the efficacy of exosomal ncRNAs as powerful biomarkers for the early diagnosis of gastric carcinomas, thereby laying a strong foundation for the advancement of novel diagnostic approaches.

## 1 Introduction

Gastric cancer (GC) ranks among the most prevalent malignant neoplasms globally. As per the latest data released by the International Agency for Research on Cancer (1, 2), the incidence of GC is the fifth highest among all malignant tumors, while its mortality rate is the fourth highest. The timely identification and management of gastric cancer are of paramount importance. However, due to the subtle nature of its early manifestations, a considerable percentage of patients get diagnosed during the intermediate or advanced stages. In cases where a patient is identified with GC at an advanced stage, the 5-year survival rate is notably low, typically below 10%. Conversely, when GC is detected in its early stages, the 5-year survival rate can be significantly higher, reaching up to 85% (3). The discovery of novel biomarkers holds substantial importance for the early-stage diagnosis and curative treatment of GC (4, 5). The diagnosis of GC routinely relies on gastroscopy, pathological evaluation, and imaging techniques (6), but these methods have certain limitations. For example, gastroscopy is an invasive procedure that involves obtaining mucosal tissue biopsies for pathological examination, which is not well accepted by patients (7), while imaging tests such as X-rays and CT scans have a low detection rate for early gastric cancer (8). Consequently, investigators have sought non-invasive or minimally invasive biomarkers to enhance the precision of GC detection.

In recent years, exosomes have attracted widespread attention as a new type of biomarker. Exosomes are double-layered lipid vesicles secreted by cells, serve as carriers for various molecular signals, such as nucleic acids and proteins, and play a crucial role in mediating intercellular communication (9). Studies (5, 10) have demonstrated that non-coding RNAs (ncRNAs) within exosomes, such as microRNAs (miRNAs), long non-coding RNAs (lncRNAs), and circular RNAs (circRNAs), which exist in exosomes, play a crucial role in the onset and development of GC. These ncRNAs are consistently detected in bodily fluids such as blood and gastric juice, suggesting their prospective application as indicative markers for the diagnosis of GC. For instance, certain investigations have demonstrated that serum exosomal biomarkers, including miR-1246 and lncRNA-GCI, are capable of accurately differentiating cancer patients from healthy individuals (11–13). The abundance of these exosomal ncRNAs exhibits notable variations between GC sufferers and healthy people, indicating their possible value as markers for the identification of GC (5).

While the diagnostic potential of exosomal ncRNAs in gastric cancer has shown promise, current evidence remains fragmented and inconsistent across studies, highlighting the need for a comprehensive evaluation. This meta-analysis systematically assesses the diagnostic accuracy of exosomal ncRNAs—such as miRNAs, lncRNAs, and circRNAs—as biomarkers for gastric cancer, synthesizing existing data to quantify their pooled sensitivity, specificity, and clinical utility. By consolidating these findings, we aim to establish a robust evidence base that can guide the integration of these biomarkers into early diagnostic protocols, ultimately improving patient outcomes through more reliable and timely detection.

## 2 Research materials and methodologies

### 2.1 Statement of ethics considerations

The herein research work was executed and recorded in accordance with the Preferred Reporting Items for Systematic Reviews and Meta-Analyses of Diagnostic Test Accuracy protocols (14), and the corresponding checklist is available in the **Supplementary Material**. The study has been registered in the PROSPERO database under the identification number CRD42024587170.

### 2.2 Search strategy

We began by identifying relevant search terms, including MeSH terms and keywords related to gastric tumors and exosomal non-coding RNAs. We conducted a systematic search across multiple databases including Embase, MEDLINE, PubMed, ScienceDirect, Scopus, and Web of Science, covering all available records through August 29, 2024 (see **Supplementary Table S1**). Following article collection, we excluded duplicates and studies lacking full-text access. We screened publications by title/abstract against predefined criteria, then progressed eligible studies to full-text review for final selection. This process identified the studies included in our final analysis.

### 2.3 Criteria for inclusion and exclusion parameters

Two assessors separately appraised the qualified articles, and any discrepancies were settled via comprehensive deliberation with the participation of a third adjudicator. Inclusion criteria stipulated that the studies: (1) investigated the role of exosomal ncRNAs for the diagnosis of GC with the application of blood samples; (2) confirmed the diagnosis of GC patients through histopathological examination; and (3) provided adequate data to establish a 2 × 2 contingency table, incorporating true positives (TP), false positives (FP), true negatives (TN), and false negatives (FN). The criteria for exclusion encompassed: (1) research works that failed to focus on the diagnostic efficacy of exosomal ncRNAs regarding GC; (2) research works that were of poor quality, lacking sufficient data, or exhibiting duplication; (3) meta-studies, conference abstracts, review articles, case reports, or seminar papers; (4) articles from which the four-cell table could not be derived; and (5) studies involving co-diagnosis in conjunction with other neoplasm indicators.

### 2.4 Data retrieval and quality evaluation

Investigators independently retrieved the following data from all qualified studies using standardized forms: (1) fundamental study attributes, encompassing first author, publication year, study country, journal name, ethnic background, sample quantity, cancer classification, tumor stage level, tumor grade degree, average age, gender ratio, sample category, ncRNA examination, reference gene element, detection technique, as well as (2) diagnostic performance metrics, such as TP, FP, FN, TN, sensitivity (Sen), specificity (Spe), and area under the receiver operating characteristic (ROC) curve (AUC). Data not directly accessible were gathered using GetData and Origin software. Each included study was systematically assessed and independently scored by researchers based on the updated QUADAS-2 framework (15). QUADAS-2 comprises four domains: case selection, test evaluation, gold standard, and case progression. The assessment primarily focused on bias risk and clinical relevance. Divergences were ironed out through consultations with a third-party evaluator to attain unanimity.

In this meta-analysis, a “study” was defined as an independent dataset with non-overlapping samples to avoid unit-of-analysis bias. If a single article reported multiple datasets (e.g., a discovery cohort and a separate validation cohort), each dataset was treated as an independent study. This approach ensured that the analysis was comprehensive and unbiased, providing a robust evaluation of the diagnostic performance of the studied exosomal ncRNAs in the context of cancer diagnosis.

### 2.5 Statistical analysis

A threshold effect analysis, employing Spearman’s correlation of ranks coefficient, was executed to explore the association between the logarithm of sensitivity and that of 1-specificity within the included studies. A non-significant correlation (p > 0.05) suggests the absence of a threshold effect contributing to heterogeneity. Heterogeneity due to non-threshold factors was assessed with the application of Cochran’s Q examination and the I² evaluative index. Significant heterogeneity was indicated by p ≤ 0.1 and I² ≥ 50%, while p > 0.1 and I² < 50% suggested no significant heterogeneity. The diagnostic performance evaluation indicators covered sensitivity, specificity, positive diagnostic likelihood ratio (DLR+), negative diagnostic likelihood ratio (DLR-), AUC, and diagnostic odds ratio (DOR).

we evaluated the diagnostic performance of exosomal ncRNAs for GC using a comprehensive set of metrics, including TP, FP, FN, and TN. These values were used to calculate Sensitivity (TP/(TP + FN)), Specificity (TN/(FP + TN)), and the AUC to assess overall diagnostic accuracy. Additionally, DLR+ and DLR− were calculated to quantify the impact of positive and negative test results on disease probability, with DLR+ = Sensitivity/(1 − Specificity) and DLR− = (1 − Sensitivity)/Specificity. DOR was also determined using the formula DOR = (TP/FN)/(FP/TN) to provide a single measure of diagnostic efficacy. These metrics collectively highlight the robustness of exosomal ncRNAs as potential biomarkers for GC diagnosis, emphasizing their ability to accurately distinguish between patients with and without gastric cancer.

For the intention of probing into the sources of heterogeneity, both meta-regression and subgroup analyses were executed. Forest plots were generated for each evaluation metric, the assessment of publication bias was appraised by means of Deek’s funnel diagram and the evaluation of clinical effectiveness was evaluated through Fagan diagrams and likelihood scatter diagrams. Meta-Disc 1.4 and Stata 17.0 software were utilized to conduct the analysis.

## 3 Results

### 3.1 Literature screening results

**Figure 1:**
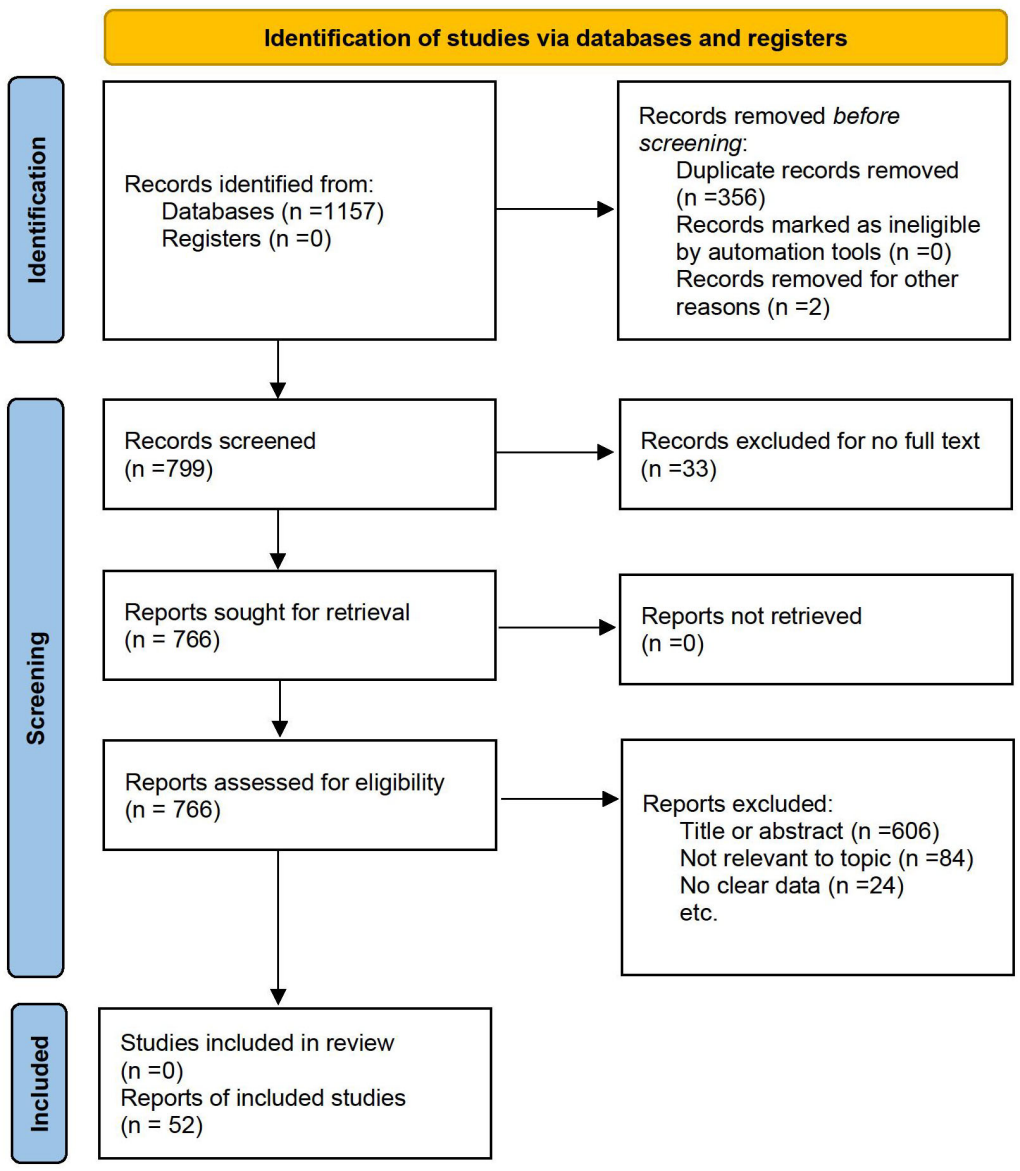
Flow chart of literature screening.

The process of identifying suitable studies is illustrated in **Figure 1**. An exhaustive literature search was performed across six databases, resulting in a total of 1157 articles. Following the removal of duplicate entries and articles for which full texts were unavailable, 766 articles were subjected to further review. Ultimately, after evaluating the titles, abstracts, and full contents of these studies, 52 articles fulfilled the eligibility requirements and were incorporated into the meta-analysis. Thus, a sum of 52 eligible articles were brought into the meta-analysis.

### 3.2 Basic details of the incorporated literatures

A total of 52 articles encompassing 164 studies were incorporated into the analysis. The included studies were conducted mainly in China, while the remaining two studies were conducted in Iran and Colombia, respectively. Specifically, 21 articles focusing on miRNAs (11, 16–35) comprised 84 studies, 17 articles on lncRNAs (13, 36–51) (37–53) included 60 studies, and 14 articles on circRNAs (52–65) involved 20 studies. The diagnostic performance metrics reported consisted of TP, FP, FN, TN, Sen, Spe, AUC, DLR+, DLR-, and DOR. Furthermore, with the aim of ensuring that the ncRNAs were sourced from exosomes, the exosomes were characterized using nanoparticle tracking assay (NTA), transmission electron microscopy (TEM), and detection of exosome-marker proteins. An overview of exosome-related information across the studies is presented in **Supplementary Table S2**. The vast majority of articles gave direct access to specific data, while the specificity and sensitivity of the remaining 17 articles were derived by analyzing the ROC curves using digital software such as GetData (11, 19–23, 25, 26, 32, 35, 38, 49, 51, 52, 58, 60, 64). The findings are detailed in **Table 1**; **Supplementary Table S3**.

**Table 1:** Basic features of the included literature.

Author	Year	Country	RNA	RNA detection	QUADAS score
miRNA					
D. Yu (16)	2024	China	miR-223-3p,miR425-5p,miR-223-3p+miR425-5p	qRT-PCR	7
H. Kahroba (17)	2022	Iran	miR-10a-5p,miR-18a-5p,miR-19b-3p,miR-215-5p,miR-10a-5p+miR-18a-5p+miR-19b-3p+miR-215-5p	RT-PCR	7
H. Yang (18)	2018	China	miR-423-5p	RT-PCR	7
J. He (19)	2023	China	miR-31,miR-192,miR-375,miR-31+miR-192+miR-375	qRT-PCR	7
J. Wang (20)	2024	China	miR-21-5p,miR-26a-5p,miR-27a-3p	qRT-PCR	7
J. Wang (21)	2022	China	miR–10401–3p,miR–1255–5p,miR–6736–5p	qRT-PCR	7
J. Yang (22)	2021	China	miR-195-5p,miR-211-5p,miR-195-5p+miR-211-5p,	qRT-PCR	7
L. Chang (23)	2021	China	miR-1228	RT-PCR	7
L. Ge (24)	2020	China	miR-1307-3p,piR-019308,piR-004918,piR-018569	qRT-PCR	6
N. Wang (25)	2017	China	miR-106a-5p,miR-19b-3p,miR-30a-5p+miR-17-5p	qRT-PCR	7
R. Ji (26)	2019	China	miR-374a-5p	qRT-PCR	6
S. Tang (27)	2020	China	miR-9-5p,let-7g-5p,let-7c-5p,miR-146b-5p,miR-92b-3p,miR-101-3p,miR-21-5p,miR-26a-5p,miR-92b-3p+let-7g-5p,miR-92b-3p+miR-146b-5p,miR-146b-5p+miR-9-5p,miR-92b-3p+miR-146b-5p+miR-9-5p	qRT-PCR	7
S. Wei (28)	2020	China	MiR-15b-3p	qRT-PCR	8
X. Lu (29)	2021	China	miR-92a-3p	qRT-PCR	6
X. Zhou (30)	2015	China	miR-185+miR-20a+miR-210+miR-25+miR-92b	qRT-PCR	7
Y. Shi (11)	2020	China	miR-1246	qRT-PCR	8
Yun. Zhang (31)	2021	China	miR-215-5p	qRT-PCR	7
Z. Huang (32)	2017	China	miR10b-5p+miR132-3p+miR185-5p+miR195-5p+miR20a3p+miR296-5p	qRT-PCR	7
Z. Ren (33)	2022	China	hsa-miR-1273g-3p,hsa-miR-619-5p,hsa-miR-4793-3p	RT-PCR	6
Z. Wang (34)	2023	China	NSD1,FBXO7,NSD1+FBXO7	RT-qPCR	7
A. Rincón-Riveros (35)	2023	Colombia	hsa-miR-451a+hsa-miR-126-3p+hsamiR-92a-3p	RNA-seq	6
LncRNA					
B. Xia (36)	2023	China	LINC00691	qRT-PCR	7
C. Cai (37)	2019	China	Lnc RNA psck2-2:1	RT-qPCR	8
C. Zhang (38)	2023	China	CCAT1	qRT-PCR	8
H. Piao (39)	2020	China	CEBPA-AS1	RT-qPCR	8
H. Xu (51)	2020	China	MIAT	qRT-PCR	7
H. Zhou (40)	2020	China	lncRNA H19	PCR	7
K. Xiao (41)	2021	China	lncRNA CCAT1	RT-qPCR	7
L. Lin (42)	2018	China	lncUEGC1,lncUEGC2	qPCR	7
L. Pan (43)	2017	China	ZFAS1	qRT-PCR	7
P. Zheng (44)	2020	China	lnc-SLC2A12-10:1	qRT-PCR	8
Q. Li (45)	2015	China	LINC00152	qRT-PCR	7
R. Zhao (46)	2018	China	HOTTIP	RT-qPCR	8
S. Li (47)	2020	China	lnc-GNAQ-6:1	RT-qPCR	8
X. Guo (48)	2023	China	lncRNA GClnc1	qRT-PCR	7
X. Guo (13)	2020	China	lncRNA-GC1	RT-PCR	7
X. Zhang (49)	2018	China	UFC1	qRT-PCR	7
Y. Zhang (50)	2021	China	FRLnc1	qRT-PCR	7
CircRNA					
J. You (52)	2023	China	circ_0001789	RT-qPCR	6
K. Xiao (53)	2022	China	Chr10q11,Chr1p11,Chr7q11,Chr10q11+Chr1p11+Chr7q11	RT-qPCR	6
P. Zheng (54)	2022	China	hsa_circ_0015286	qRT-PCR	8
R. Li (55)	2023	China	CircRNA CDR1as	RT-qPCR	8
W. Tang (56)	2018	China	circ-KIAA1244	qRT-PCR	7
X. Huang (57)	2023	China	Hsa_circ_000200	qRT-PCR	7
X. Li (58)	2023	China	hsa_circ_0079439	dd-PCR	7
X. Tao (59)	2020	China	Hsa_circ_0000419	qRT-PCR	6
X. Yang (60)	2023	China	circLPAR1	RT-PCR	7
X. Zang (61)	2024	China	circ50547	qRT-PCR	7
Y. Shao (62)	2020	China	hsa_circ_0065149	qRT-PCR	8
Y. Wang (63)	2021	China	CircITCH	qRT-PCR	7
Z. Zhang (64)	2022	China	circFCHO2	qRT-PCR	6
X. Sun (65)	2022	China	Hsa_circ_0002874	RT-PCR	8

### 3.3 Evaluation of research quality

The results obtained from the QUADAS-2 quality assessment of the 52 incorporated articles are demonstrated in **Supplementary Figure S1**, with the QUADAS scores being shown in **Table 1**. The results of each study were assessed as “yes”, “no” or “unclear”. Specifically, “yes” was assigned 1 point, “no” was assigned 1 point and “unclear” was assigned 0 points. Studies with a score exceeding 4 were defined by us as high-quality studies, while those with a score below 4 were considered as low-quality studies. The overall quality of the included literature was satisfactory, and all of them were of high quality.

### 3.4 Heterogeneity analysis

Threshold effects represent fundamental sources of heterogeneity in diagnostic tests. Therefore, for diagnostic meta-analyses, the presence of threshold effects is initially evaluated. The Spearman correlation coefficients, calculated between the natural logarithms of sensitivity and specificity using Meta DiSc 14.0 software, were 0.426, 0.172, and 0.722 for exosomal miRNAs, lncRNAs, and circRNAs studies, respectively. Corresponding p-values were p < 0.001, p = 0.188 (p > 0.05), and p < 0.001, respectively. These findings indicate that threshold effects contribute to heterogeneity in exosomal miRNAs and circRNAs studies but not in lncRNAs studies. Heterogeneity across the included studies was further assessed using the Cochran’s Q test and the I² statistic in Stata software. For miRNAs, lncRNAs, and circRNAs, the results were: Cochran’s Q = 241.07 (p < 0.001), I² = 65.6% (95% CI, 49.8–74.9); Cochran’s Q = 425.58 (p < 0.001), I² = 86.1% (95% CI, 77.1–90.7); and Cochran’s Q = 62.85 (p < 0.001), I² = 69.8% (95% CI, 22.3–84.0), respectively. These results demonstrate significant heterogeneity among studies, attributable to factors other than threshold effects.

### 3.5 Diagnostic accuracy evaluation of exosomal ncRNAs

**Figure 2:**
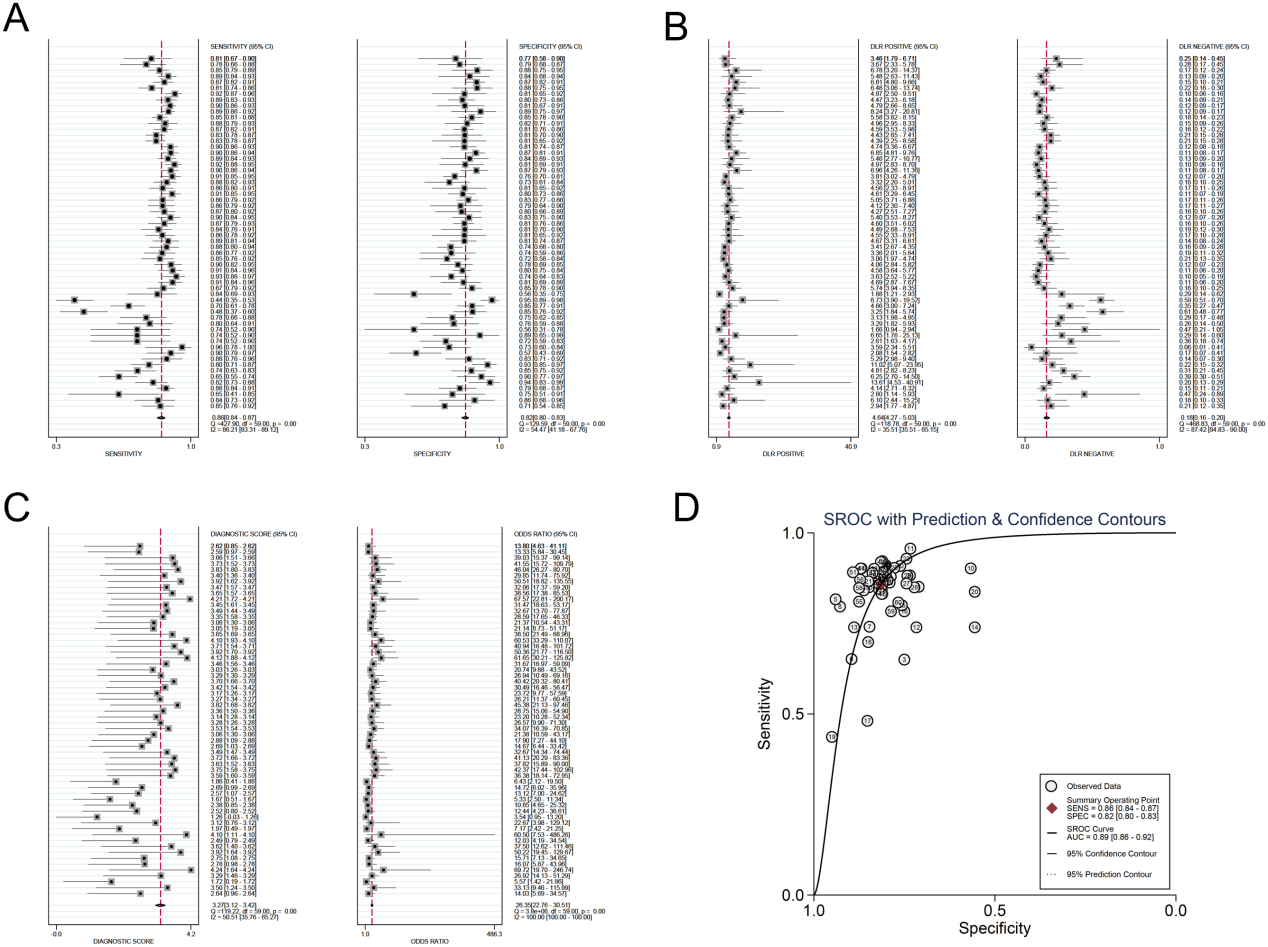
Diagnostic efficacy of exosomal lncRNA in gastric cancer patients. **(A)** Sensitivity and specificity. **(B)** Diagnostic likelihood ratios. **(C)** Diagnostic score and odds ratio. **(D)** SROC curve.

**Figure 3:**
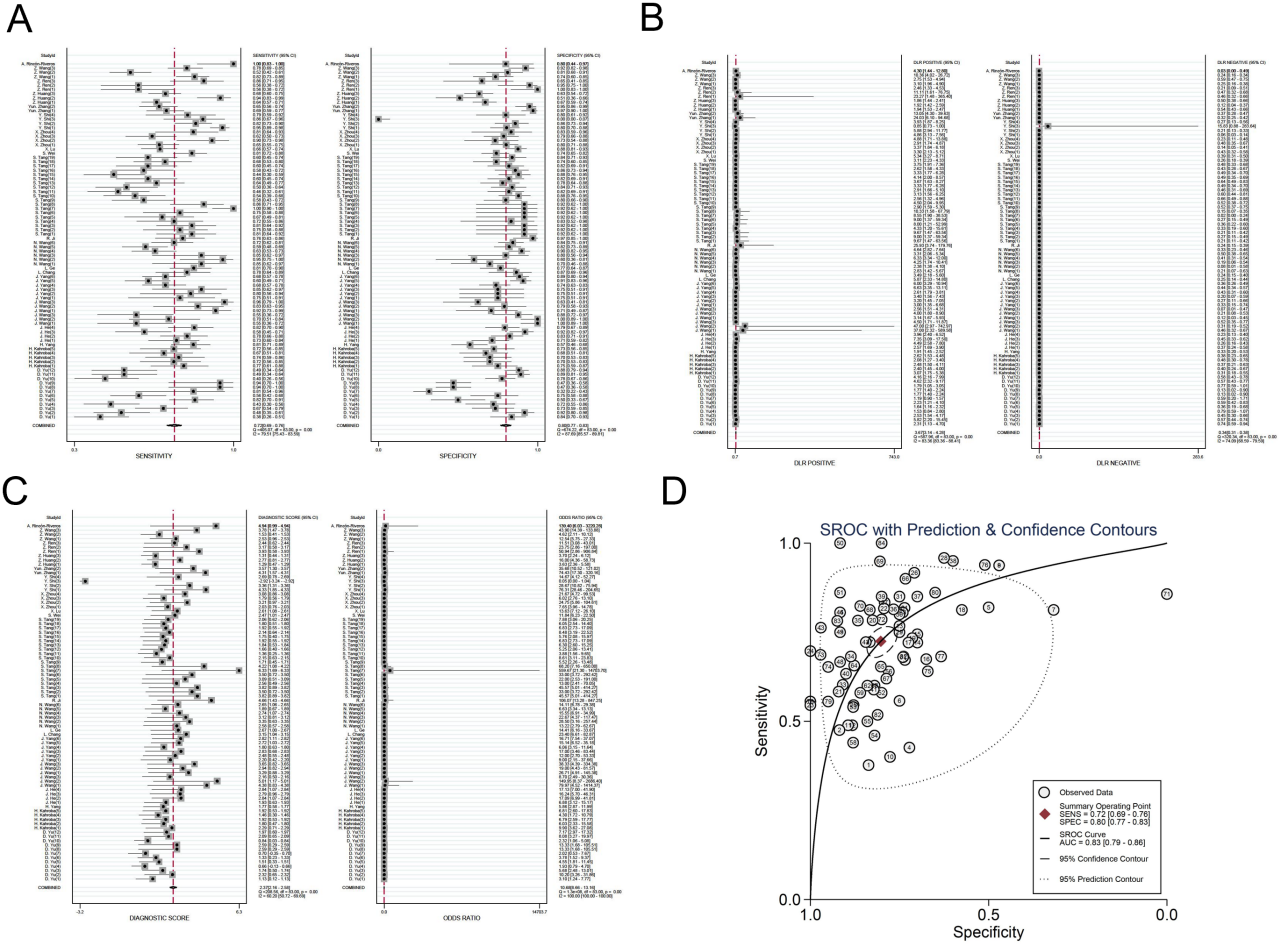
Diagnostic efficacy of exosomal miRNA in gastric cancer patients. **(A)** Sensitivity and specificity. **(B)** Diagnostic likelihood ratios. **(C)** Diagnostic score and odds ratio. **(D)** SROC curve.

**Figure 4:**
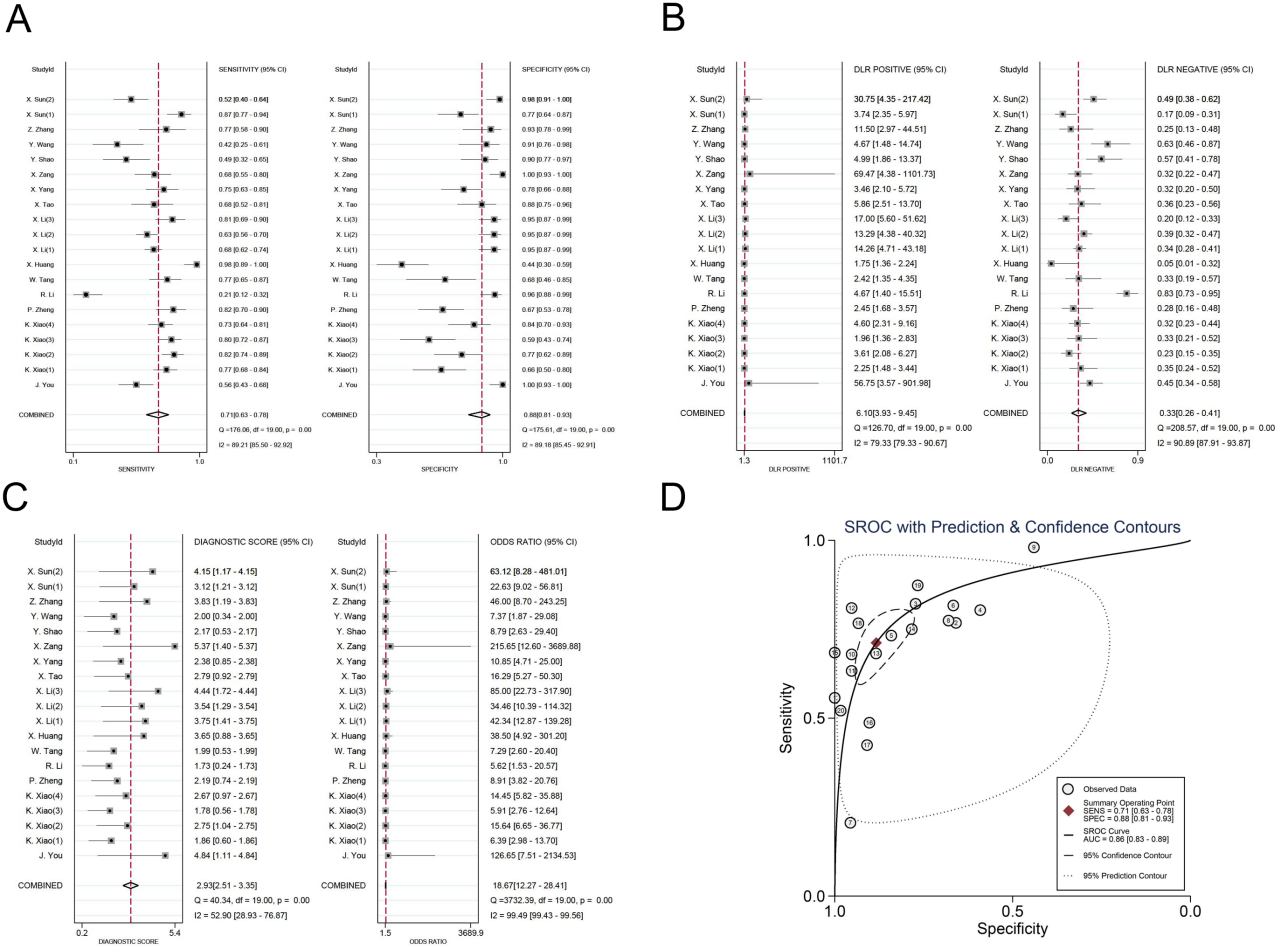
Diagnostic efficacy of exosomal circRNA in gastric cancer patients. **(A)** Sensitivity and specificity. **(B)** Diagnostic likelihood ratios. **(C)** Diagnostic score and odds ratio. **(D)** SROC curve.

The investigators employed a random effects model to assess the diagnostic impact of exosomal miRNAs, lncRNAs, and circRNAs in GC. The overall sensitivity and specificity derived from the lncRNAs studies were 0.86 (95% CI, 0.84–0.87) and 0.82 (95% CI, 0.80–0.83), respectively. The aggregated diagnostic score and DOR were calculated as 3.27 (95% CI, 3.12 to 3.42) and 26.35 (95% CI, 22.76 to 30.51), respectively. A forest plot illustrating these findings is presented in **Figure 2** from A to C. The included studies exhibited substantial heterogeneity in terms of sensitivity (p ≤ 0.001, I^2^ = 86.21%), specificity (p ≤ 0.001, I^2^ = 54.47%), DLR+ (p ≤ 0.001, I^2^ = 35.51%), DLR- (p ≤ 0.001, I^2^ = 87.42%), diagnostic score (p ≤ 0.001, I^2^ = 50.51%), and DOR (p ≤ 0.001, I^2^ = 100%). A summary receiver operating characteristic (SROC) curve was constructed, as depicted in **Figure 2D**, yielding an overall AUC of 0.89 (95% CI, 0.86–0.92). Furthermore, SROC curves for exosomal miRNAs and circRNAs were generated using a random effects model to evaluate their diagnostic potential in GC. The respective AUC were 0.83 (95% CI, 0.79–0.86) and 0.87 (95% CI, 0.84–0.90), as shown in **Figure 3** from A to D and **Figure 4** from A to D. By comparing high-frequency exosomal ncRNAs, lncRNA was found to be the most consistent biomarker, as shown in **Supplementary Table S4**, which suggests that among these ncRNAs, lncRNA has the most consistent diagnostic value in gastric cancer diagnosis.

### 3.6 Meta-analysis and subgroup analysis

Given the observed heterogeneity, we conducted meta-regression and subgroup analyses to evaluate miRNAs, lncRNAs, and circRNAs. These analyses were performed to examine the influence of control source, exosome extraction method, and RNA detection method on miRNAs, lncRNAs, and circRNAs, considering factors such as sample size, sample type, control source, exosome extraction method, and RNA detection. The findings are summarized in **Table 2**. The meta-regression results indicated significant heterogeneity among studies investigating exosomal miRNAs, lncRNAs, and circRNAs. Specifically, the heterogeneity in exosomal miRNAs for diagnosing GC was influenced by sample size (p = 0.045), exosome extraction method (p = 0.001), and RNA detection method (p = 0.013). In contrast, the type of control (p = 0.555), sample type (p = 0.458), and the number of miRNAs detections (p = 0.173) did not significantly affect the overall outcomes. Subgroup analysis revealed that exosomal miRNAs derived from plasma exhibited higher sensitivity compared to those from serum. Additionally, exosomes isolated using exosome extraction kits demonstrated superior diagnostic performance relative to those obtained via ultracentrifugation. Furthermore, miRNAs detected by reverse transcription-polymerase chain reaction (RT-PCR) showed better diagnostic performance than those identified using quantitative real-time polymerase chain reaction (QRT-PCR).

**Table 2:** Results of subgroup analysis.

Subgroup	Number of studies	Sensitivity [95%CI]	Specificity [95%CI]	DLR +[95%CI]	DLR -[95%CI]	AUC [95%CI]	DOR [95%CI]	P Value
miRNA								
Sample size								0.045
<100	39	0.79 [0.74-0.83]	0.81 [0.73-0.87]	4.07 [2.92-5.68]	0.26 [0.22-0.32]	0.86 [0.82-0.89]	15.46 [10.10-23.66]	
≥100	45	0.67 [0.63-0.71]	0.81 [0.77-0.84]	3.56 [3.02-4.20]	0.40 [0.36-0.46]	0.81 [0.77-0.84]	8.80 [7.02-11.03]	
Types of sample								0.458
Serum	69	0.72 [0.68-0.76]	0.80 [0.76-0.84]	3.66 [3.03-4.42]	0.35 [0.31-0.40]	0.82 [0.79-0.85]	10.45 [8.13-13.43]	
Plasma	15	0.74 [0.68-0.79]	0.80 [0.76-0.84]	3.77 [3.11-4.58]	0.32 [0.26-0.40]	0.84 [0.81-0.87]	11.66 [8.60-15.81]	
Types of contrast								0.555
HC	71	0.72 [0.69-0.76]	0.81 [0.78-0.84]	3.82 [3.26-4.48]	0.34 [0.30-0.38]	0.83 [0.80-0.86]	11.24 [9.40-13.90]	
Patients	6	0.73 [0.53-0.87]	0.78 [0.63-0.88]	3.31 [1.83-6.01]	0.34 [0.18-0.65]	0.83 [0.79-0.86]	9.68 [3.28-28.55]	
MIX	7	0.73 [0.52-0.87]	0.73 [0.51-0.88]	2.70 [1.51-4.86]	0.37 [0.22-0.64]	0.79 [0.75-0.82]	7.24 [3.13-16.75]	
Methods of exosome extraction								0.001
exosome isolation kit	57	0.75 [0.71-0.79]	0.81 [0.77-0.84]	3.86 [3.22-4.63]	0.31 [0.27-0.36]	0.85 [0.81-0.87]	12.50 [9.70-16.12]	
Ultracentrifugation	21	0.65 [0.57-0.72]	0.77 [0.69-0.84]	2.85 [2.16-3.76]	0.46 [0.39-0.54]	0.76 [0.72-0.80]	6.25 [4.45-8.79]	
Other	6	0.70 [0.56-0.81]	0.87 [0.73-0.94]	5.38 [2.66-10.91]	0.35 [0.24-0.50]	0.85 [0.82-0.88]	15.53 [6.99-34.53]	
RNA detection								0.013
QRT-PCR	77	0.72 [0.68-0.75]	0.81 [0.77-0.84]	3.82 [3.23-4.53]	0.35 [0.31-0.39]	0.83 [0.80-0.86]	11.07 [8.83-13.87]	
RT-PCR	6	0.75 [0.69-0.80]	0.68 [0.61-0.74]	2.32 [1.90-2.84]	0.37 [0.30-0.46]	0.78 [0.74-0.81]	6.21 [4.28-9.01]	
RNA-seq	1	/	/	/	/	/	/	
Number of miRNA detected								0.173
Single	58	0.72 [0.68-0.77]	0.82 [0.77-0.86]	3.97 [3.16-5.00]	0.33 [0.29-0.38]	0.83 [0.80-0.86]	11.93 [8.89-16.01]	
Mutiple	26	0.72 [0.65-0.77]	0.78 [0.73-0.82]	3.20 [2.70-3.79]	0.37 [0.31-0.44]	0.82 [0.78-0.85]	8.73 [6.64-11.47]	
LncRNA								
Sample size								0.000
<100	9	0.80 [0.76-0.84]	0.73 [0.68-0.78]	3.00 [2.46-3.66]	0.27 [0.21-0.34]	0.84 [0.80-0.87]	11.18 [7.64-16.35]	
≥100	51	0.86 [0.84-0.88]	0.82 [0.80-0.84]	4.83 [4.45-5.24]	0.17 [0.15-0.19]	0.90 [0.87-0.92]	28.57 [24.81-32.91]	
Types of sample								0.916
Serum	22	0.83 [0.79-0.86]	0.85 [0.81-0.87]	5.38 [4.54-6.37]	0.20 [0.16-0.25]	0.90 [0.88-0.93]	26.66 [20.90-34.01]	
Plasma	38	0.87 [0.85-0.89]	0.80 [0.78-0.81]	4.27 [3.91-4.65]	0.16 [0.14-0.19]	0.88 [0.85-0.90]	26.11 [21.54-31.64]	
Types of contrast								0.834
HC	26	0.85 [0.81-0.88]	0.82 [0.79-0.85]	4.67 [4.02-5.42]	0.18 [0.15-0.22]	0.90 [0.87-0.92]	25.48 [19.95-32.55]	
Patients	23	0.87 [0.84-0.89]	0.81 [0.78-0.83]	4.47 [3.94-5.07]	0.17 [0.14-0.19]	0.88 [0.84-0.90]	26.94 [21.87-33.18]	
MIX	11	0.85 [0.79-0.89]	0.82 [0.78-0.84]	4.61 [3.97-5.36]	0.18 [0.13-0.26]	0.88 [0.85-0.91]	24.95 [17.14-36.31]	
Methods of exosome extraction								0.108
exosome isolation kit	38	0.86 [0.84-0.88]	0.81 [0.79-0.82]	4.48 [4.14-4.86]	0.17 [0.15-0.20]	0.87 [0.84-0.90]	26.13 [21.83-31.29]	
Ultracentrifugation	13	0.85 [0.79-0.89]	0.85 [0.82-0.88]	5.66 [4.79-6.69]	0.18 [0.13-0.24]	0.91 [0.88-0.93]	31.65 [23.99-41.76]	
Other	9	0.82 [0.78-0.86]	0.77 [0.68-0.84]	3.65 [2.59-5.14]	0.23 [0.18-0.28]	0.85 [0.82-0.88]	16.12 [10.04-25.87]	
RNA detection								0.002
QRT-PCR	43	0.85 [0.83-0.87]	0.82 [0.80-0.83]	4.65 [4.27-5.06]	0.18 [0.16-0.21]	0.89 [0.86-0.91]	25.65 [21.69-30.32]	
RT-PCR	10	0.88 [0.86-0.89]	0.84 [0.81-0.87]	5.60 [4.73-6.63]	0.15 [0.13-0.17]	0.92 [0.89-0.94]	38.23 [30.34-48.17]	
Other(PCR, QPCR)	7	0.83 [0.75-0.88]	0.75 [0.66-0.82]	3.31 [2.42-4.53]	0.23 [0.17-0.32]	0.86 [0.83-0.89]	14.25 [8.66-23.46]	
Number of lncRNA detected								/
Single	60	0.86 [0.84-0.87]	0.82 [0.80-0.83]	4.64 [4.27-5.03]	0.18 [0.16-0.20]	0.89 [0.86-0.92]	26.35 [22.76-30.51]	
Mutiple	/	/	/	/	/	/	/	
CircRNA								
Sample size								0.243
<100	5	0.63 [0.50-0.75]	0.88 [0.79-0.93]	5.07 [3.20-8.04]	0.42 [0.31-0.57]	0.85 [0.82-0.88]	12.11 [6.98-20.99]	
≥100	15	0.73 [0.63-0.81]	0.89 [0.79-0.94]	6.47 [3.59-11.68]	0.30 [0.23-0.40]	0.87 [0.84-0.90]	21.46 [12.56-36.69]	
Types of sample								0.304
Serum	8	0.75 [0.63-0.84]	0.82 [0.65-0.92]	4.212.25-7.87]	0.30 [0.22-0.42]	0.85 [0.81-0.88]	14.05 [7.93-24.89]	
Plasma	12	0.68 [0.57-0.77]	0.91 [0.84-0.95]	7.64 [4.44-13.13]	0.35 [0.27-0.47]	0.87 [0.84-0.90]	21.54 [12.26-37.84]	
Types of contrast								0.137
HC	17	0.71 [0.61-0.79]	0.89 [0.81-0.94]	6.28 [3.90-10.13]	0.32 [0.25-0.43]	0.87 [0.84-0.90]	19.40 [12.05-31.22]	
Patients	1	/	/	/	/	/	/	
MIX	2	/	/	/	/	/	/	
Methods of exosome extraction								0.257
exosome isolation kit	11	0.68 [0.52-0.81]	0.89 [0.78-0.95]	5.98 [3.31-10.79]	0.36 [0.24-0.54]	0.87 [0.83-0.89]	16.58 [9.13-30.12]	
Other	9	0.74 [0.67-0.80]	0.88 [0.76-0.94]	6.18 [3.18-12.01]	0.30 [0.25-0.35]	0.84 [0.80-0.87]	20.90 [11.33-38.55]	
RNA detection								0.000
QRT-PCR	12	0.68 [0.53-0.81]	0.90 [0.80-0.95]	6.98 [3.76-12.93]	0.35 [0.24-0.52]	0.88 [0.85-0.90]	19.96 [10.95-36.37]	
RT-PCR	5	0.78 [0.74-0.81]	0.74 [0.65-0.81]	2.94 [2.19-3.95]	0.30 [0.25-0.36]	0.81 [0.77-0.84]	9.70 [6.39-14.72]	
Other	3	/	/	/	/	/	/	
Number of circRNA detected								0.847
Single	19	0.71 [0.62-0.79]	0.89 [0.81-0.93]	6.24 [3.90-9.97]	0.33 [0.25-0.42]	0.86 [0.83-0.89]	19.11 [12.17-30.00]	
Mutiple	1	/	/	/	/	/	/	

For lncRNAs, subgroup analysis was conducted to evaluate the impact of various variables. The heterogeneity in exosomal lncRNAs for GC diagnosis was associated with sample size (p < 0.001) and lncRNA detection method (p = 0.002). However, sample type (p = 0.916), control source (p = 0.834), and exosome extraction method (p = 0.108) did not significantly influence the diagnostic outcomes.

Regarding circRNAs, the inter-study heterogeneity in gastric cancer diagnosis was not related to sample size (p = 0.243), sample type (p = 0.304), control type (p = 0.137), exosome extraction method (p = 0.257), or the number of circRNAs tests (p = 0.847). Instead, the heterogeneity was primarily attributed to the RNA detection method (p < 0.001). Subgroup analysis demonstrated that samples with a size exceeding 100 exhibited enhanced diagnostic sensitivity and specificity. Moreover, circRNAs detected by QRT-PCR showed improved diagnostic specificity, while those identified by RT-PCR exhibited superior diagnostic sensitivity.

### 3.7 Publication bias

Publication bias was assessed across the included studies through the application of the Deek funnel plot asymmetry test, as illustrated in **Supplementary Figure S2**. The analysis revealed no evidence of potential publication bias for circRNAs (p = 0.46). However, miRNAs (p = 0.04) and lncRNAs (p < 0.001) demonstrated statistically significant results, suggesting the presence of potential publication bias in these categories.

### 3.8 Clinical significance

To investigate the clinical relevance of miRNAs, lncRNAs, and circRNAs in the diagnosis of GC, we developed a Fagan diagram to illustrate the relationships among pre-test probability, likelihood ratios, and post-test probability. As depicted in **Supplementary Figure S3**, for exosomal miRNAs, with an assumed pre-test probability of 50%, the post-test probability reaches 79% based on the DLR+, suggesting that exosomal miRNAs exhibit strong clinical utility in diagnosing GC. Similarly, for exosomal lncRNAs, a pre-test probability of 50% yields a post-test probability of 82%, indicating that exosomal lncRNAs also demonstrate robust diagnostic potential for GC. For exosomal circRNAs, a pre-test probability of 50% results in a post-test probability of 85%, highlighting the significant diagnostic value of exosomal circRNAs in GC.

Additionally, a likelihood ratio scatter plot analysis was conducted. The scatter plot is segmented into four quadrants: the upper left quadrant (LUQ), upper right quadrant (RUQ), lower left quadrant (LLQ), and lower right quadrant (RLQ). In the LUQ, where DLR+ exceeds 10 and DLR- is below 0.1, the test is capable of both confirming and excluding gastric cancer. In the RUQ, with DLR+ greater than 10 and DLR- above 0.1, the test can only confirm gastric cancer. In the LLQ, where DLR+ is less than 10 and DLR- is below 0.1, the test is limited to excluding gastric cancer. In the RLQ, with DLR+ less than 10 and DLR- greater than 0.1, the test fails to either confirm or exclude gastric cancer.

As illustrated in **Supplementary Figure S4**, the diagnostic performance of exosomal miRNAs, lncRNAs, and circRNAs in both diagnosing and excluding GC is relatively similar. The likelihood ratio plot reveals that the summary point of the positive likelihood ratio (PLR) and negative likelihood ratio (NLR) falls within the lower right quadrant, indicating that exosomal miRNAs, lncRNAs, and circRNAs are not suitable as standalone diagnostic tools for either confirming or ruling out GC.

## 4 Disscussion

GC remains a significant global health challenge with a 5-year survival rate of only 20-30%, particularly for patients diagnosed at advanced stages (66). The poor prognosis and high mortality of GC are largely attributed to the lack of highly sensitive, specific and efficient early diagnostic strategies (7). Recent advances in liquid biopsy based on non-coding RNAs (ncRNAs), including miRNAs, lncRNAs and circRNAs, have shown great promise in addressing this issue (67). Exosomal ncRNAs, such as miR-1246, lncRNA GClnc1 and circ50547, have demonstrated high diagnostic accuracy and prognostic potential in GC, reflecting tumour heterogeneity and enabling non-invasive detection by blood tests (11, 13, 61). China has emerged as a leading contributor in exosomal ncRNA research for GC diagnosis, driven by its high epidemiological burden of GC and extensive clinical resources (68). In summarising the meta-analysis studies, we found that Chinese researchers have made full use of the rich clinical sample resources, combined with advanced technologies such as liquid biopsy and high-throughput sequencing, to publish a large number of high-quality research results. However, significant contributions have also come from other regions, including Japan, USA and Western countries, which have enriched our understanding of the molecular mechanisms and clinical applications of exosomal ncRNAs (69, 70). Exosomes are membrane-bound vesicles released following the fusion of multivesicular bodies with the cell membrane. These vesicles, approximately 30-150 nm in diameter, contain a variety of components, including proteins, lipids, nucleic acids, and metabolites (71, 72). Exosomes facilitate normal cellular functions, such as activating T cells during immune responses (72), and also contribute to tumor progression (73). Tumor-derived exosomes can transmit pro-tumorigenic signals, promoting cell proliferation, migration, invasion, and angiogenesis (74).

R. Ji et al. (26) demonstrated that exosomal miR-374a-5p achieved an AUC of 0.919 (95% CI: 0.866-0.972) in diagnosing GC, highlighting its high diagnostic accuracy, and the sensitivity was 0.761, meaning that this marker could correctly identify 76.1% of gastric cancer patients, which is slightly lower than the specificity, but still of high clinical value, especially in early diagnosis. The specificity was as high as 0.965, indicating that miR-374a-5p performed well in identifying non-gastric cancer patients with a very low misdiagnosis rate, which is important for reducing unnecessary treatment and psychological burden. In addition, miR-374a-5p had a low false positive rate (FP = 1), further confirming its reliability in diagnosis. Notably, miR-374a-5p exhibited a DOR of 102.398, outperforming other miRNAs such as miR-223-3p and miR-425-5p (16). Furthermore, miR-374a-5p, which is overexpressed in GC patients, was significantly associated with chemoresistance, suggesting its dual role as a diagnostic biomarker and a predictor of treatment response. These findings position miR-374a-5p as a promising non-invasive tool for GC diagnosis and therapeutic guidance.

X. Guo et al. (13) investigated exosomal lncRNA-GC1, which exhibits elevated expression in individuals with early-stage GC. The diagnostic performance of exosomal lncRNA-GC1, assessed in both the combined test set and the validation set, demonstrated a sensitivity of 0.8824, a specificity of 0.8229, and an AUC of 0.8905 (95% CI: 0.8371–0.9438) for detecting early-stage GC. Similarly, the aggregated sensitivity, specificity, and AUC values for lncRNAs across the 68 studies we reviewed were consistent, with respective values of 0.86, 0.82, and 0.89 (95% CI: 0.86–0.92). K. Xiao et al. (41) identified that the exosomal long non-coding RNA CCAT1 exhibited elevated expression levels in gastric cancer patients, demonstrating an AUC value of 0.89, a sensitivity of 0.80, and a specificity of 0.93. These findings underscore the potential of exosomal lncRNAs, such as lncRNA-GC1 and CCAT1, as reliable biomarkers for the early detection of GC, offering high diagnostic accuracy and consistency across studies. Comparable diagnostic performance was also noted for exosomal circRNAs (58). X. Yang et al. (60) demonstrated that the expression level of exosomal circLPAR1 in the serum of gastric cancer patients was significantly decreased, exhibiting an AUC of 0.836 (95% CI: 0.765-0.906), a sensitivity of 0.748, and a specificity of 0.780. These findings underscore the potential diagnostic significance of exosomal ncRNAs in GC.

This meta-analysis reveals both the diagnostic promise and current limitations of exosomal ncRNAs for gastric cancer detection through a comprehensive evaluation of 52 studies. The findings demonstrate robust performance across ncRNA classes, with lncRNAs showing the highest diagnostic accuracy (pooled AUC 0.89; 95% CI 0.86-0.92), followed by circRNAs (0.86; 0.83-0.89) and miRNAs (0.83; 0.79-0.86). Among individual biomarkers, lncRNA GClnc1 (48) emerged as particularly noteworthy, maintaining consistent accuracy (AUC 0.84-0.94) across 28 independent studies, while miR-374a-5p (26) (AUC 0.92) and miR-1246 (11) (sensitivity 0.79-0.95) showed excellent but less validated performance in specific cohorts.

The analysis uncovered several critical insights regarding biomarker performance. Multi-marker panels surprisingly failed to outperform single biomarkers, likely due to overfitting in small discovery cohorts. Certain circRNAs, exemplified by hsa_circ_0079439 (58), demonstrated exceptional specificity exceeding 95% despite more modest sensitivity (63-81%), suggesting their potential as complementary diagnostic tools. The superior consistency observed for lncRNAs may reflect their stable packaging in exosomes and tissue-specific expression patterns.

However, substantial heterogeneity challenges the interpretation of these findings, stemming from multiple sources. The evaluation of 59 distinct miRNAs, 17 lncRNAs, and 16 circRNAs across studies, with minimal target overlap, introduces significant variability in reported diagnostic performance. Meta-regression identified technical factors contributing to this heterogeneity, particularly RNA detection methods and exosome extraction protocols, though the fundamental issue of non-overlapping ncRNA targets remains unresolved. This variability limits the generalizability of pooled results, as diagnostic accuracy often appears dependent on specific RNA targets rather than reflecting consistent biological signals.

The most promising biomarkers, including GClnc1, miR-374a-5p, lncRNA-GC1, and CCAT1, demonstrated consistently high diagnostic accuracy (AUCs 0.88-0.92) across multiple reports (13, 26, 38, 41, 48). Nevertheless, the field faces critical challenges requiring immediate attention. Standardized methodologies for exosome isolation and ncRNA detection must be developed to reduce technical variability, while consensus frameworks are needed for biomarker prioritization and validation. Future research should prioritize multicenter prospective studies to validate these candidates, addressing current limitations in study design and methodological consistency to advance the most robust biomarkers toward clinical implementation.

Our synthesis of the literature revealed an interesting pattern regarding single versus multiple ncRNA markers, though we emphasize these observations derive from cross-study comparisons rather than direct experimental comparisons. Several individual studies reported superior diagnostic performance for single ncRNA markers compared to multi-marker combinations. For example, miR-374a-5p (26) (AUC = 0.919, sensitivity = 0.761, specificity = 0.965) and lncRNA-GC1 (13) (AUC = 0.8905, sensitivity = 0.8824, specificity = 0. 8229), tended to have higher AUC and DOR than multiple marker combinations. For instance, the combination of miR-223-3p and miR-425-5p had a lower AUC (0.707) and DOR (17.191) compared to a single marker (16). However, these comparisons must be interpreted cautiously as they originate from distinct studies employing different methodologies, patient cohorts, and experimental conditions. The apparent advantage of single markers may reflect study-specific factors rather than inherent biological superiority. This observation underscores the need for future studies specifically designed to directly compare single and combinatorial ncRNA approaches within standardized experimental frameworks, which would provide more definitive evidence for optimizing diagnostic strategies.

## 5 Limitations

The research presents several limitations. Initially, the inclusion of numerous retrospective investigations may introduce selection bias. Secondly, the exclusion of non-English publications could lead to information bias, as valuable data might be overlooked. Thirdly, variations in study design and execution may introduce confounding variables, potentially influencing the outcomes. The substantial heterogeneity among the studies poses a challenge to the reliability and reproducibility of the meta-analysis findings.

A comprehensive meta-analysis encompassing numerous studies has revealed that exosomal ncRNAs are pivotal in the diagnostic process of gastric cancer. Specifically, miRNAs, lncRNAs, and circRNAs exhibit substantial clinical relevance, as evidenced by their favorable positive diagnostic likelihood ratios. To translate these findings into clinical practice, it is imperative to elucidate the underlying mechanisms through which these ncRNAs contribute to the diagnosis and physiological aspects of GC. This understanding should be substantiated through rigorous experimental validation and *in vivo* model studies.

## Data Availability

The raw data supporting the conclusions of this article will be made available by the authors, without undue reservation.
