# Supplementary material for: Diagnostic value of exosome non-coding RNAs as non-invasive biomarkers in gastric cancer: a meta-analysis
Source: Front Oncol. 2025 Jun 12;15:1570020. doi: 10.3389/fonc.2025.1570020 (PMC12197921; doi:10.3389/fonc.2025.1570020)
Supplement: Supplementary file 1 [file DataSheet1.docx]

Supplementary Material

# Supplementary Figures and Tables

## Fig. S1 Literature quality assessment.

## Fig. S2 Deek's funnel plot asymmetry test was used to detect articles publication bias. (A) Detection of publication bias in articles related to exosomal miRNA release in gastric cancer patients. P=0.04, indicating publication bias. (B) Publication bias detection of exosomal lncRNA released by gastric cancer patients. The results showed that P＜0.01, indicating publication bias. (C) Publication bias detection of circRNA-related articles on exosomes released by gastric cancer patients. The results showed that P=0.46 greater than 0.05 indicated no publication bias.

## Fig. S3 The clinical significance of exosomal non-coding RNA in the diagnosis of gastric cancer was evaluated by Fagan diagram. (A) exosomal miRNA. (B) exosomal lncRNA. (C) exosomal circRNA.

## Fig. S4 The likelihood ratio scattergram to analyze the clinical significance of exosomal non-coding RNA in the diagnosis of gastric cancer. (A)exosomal miRNA. (B)exosomal lncRNA. (C)exosomal circRNA.

## Table S1 Search Strategy

## Table S2 The information for exosomal ncRNAs.

## Table S3 Values the diagnostic effectiveness of the included studies.

Table S4 Diagnostic Performance of Frequently Studied Exosomal ncRNAs in Gastric Cancer.

## Supplementary Figures


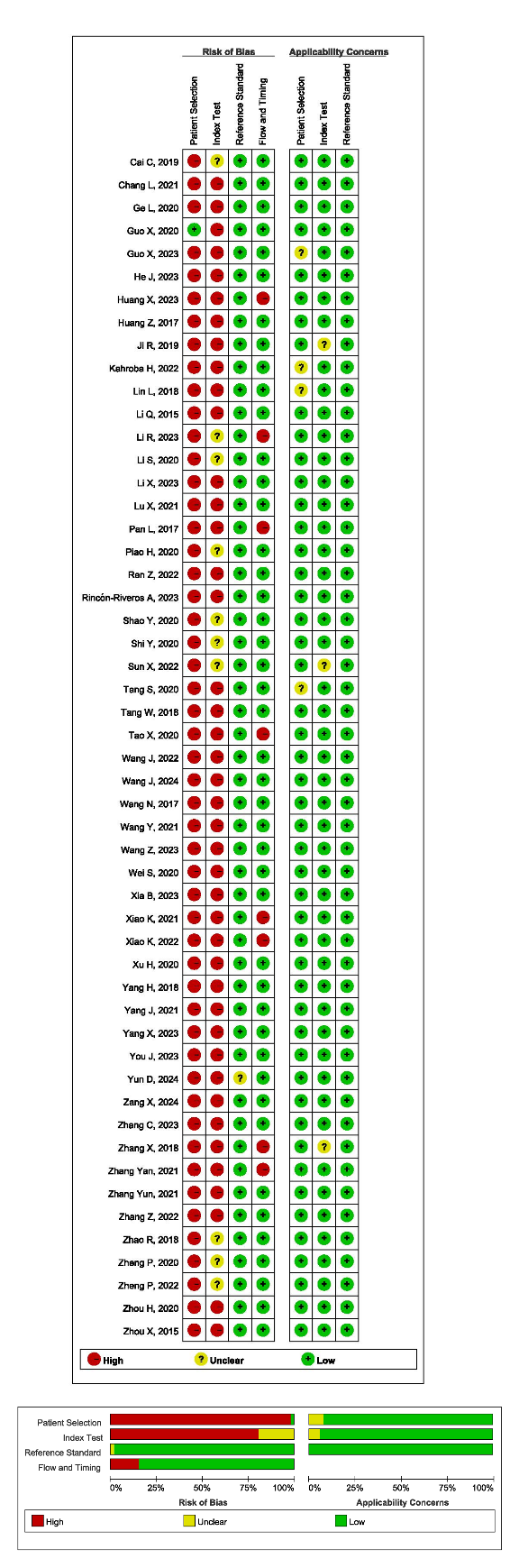


**Fig. S1 Literature quality assessment.**

**A**

**
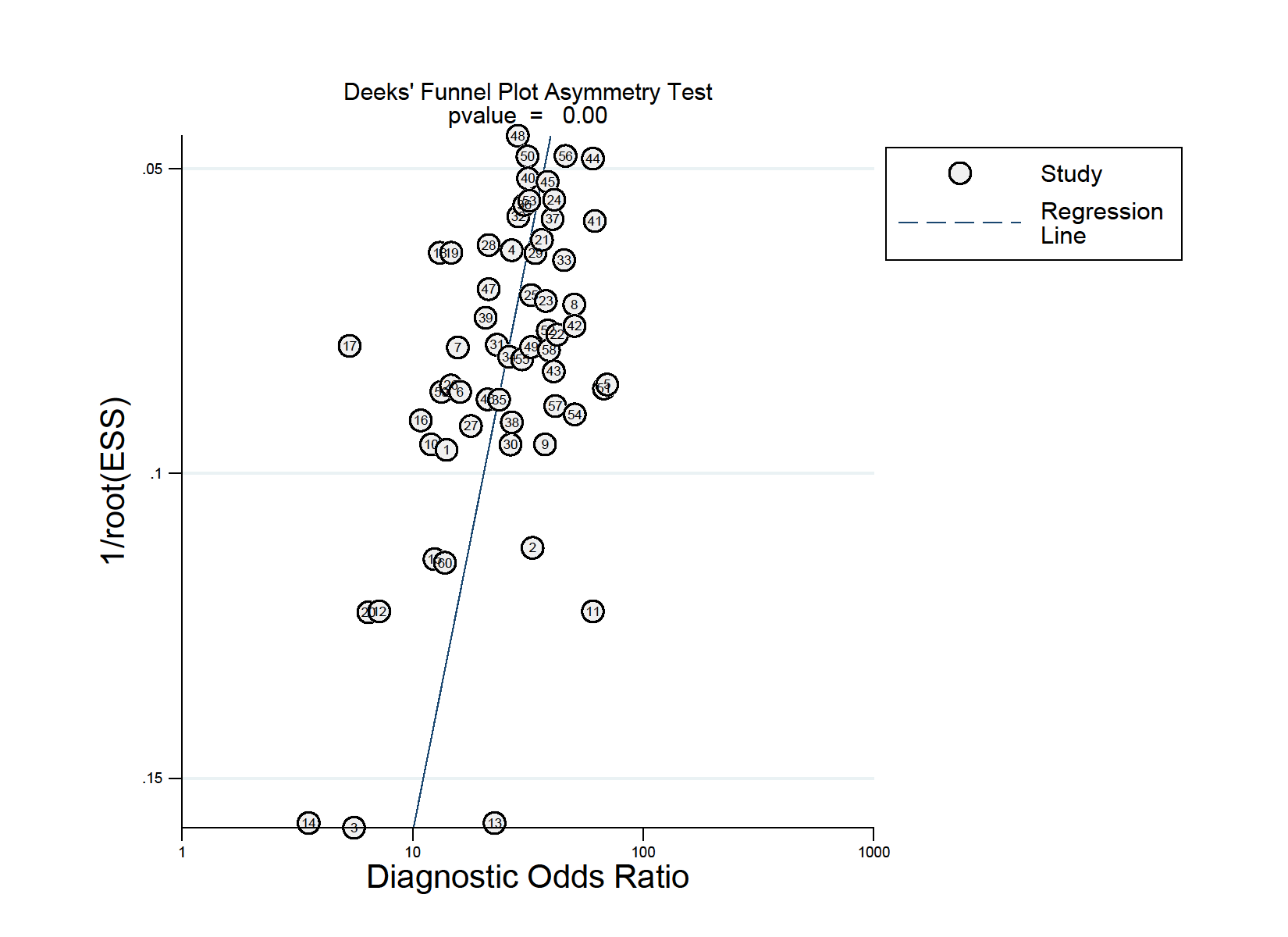
**

**Fig. S2 Deek's funnel plot asymmetry test was used to detect articles publication bias. (A)** Detection of publication bias in articles related to exosomal miRNA release in gastric cancer patients. P=0.04, indicating publication bias; **(B)** Publication bias detection of exosomal lncRNA released by gastric cancer patients. The results showed that P＜0.01, indicating publication bias; **(C)** Publication bias detection of circRNA-related articles on exosomes released by gastric cancer patients. The results showed that P=0.46 greater than 0.05 indicated no publication bias.

**B**





**Fig. S2 Deek's funnel plot asymmetry test was used to detect articles publication bias. (A)** Detection of publication bias in articles related to exosomal miRNA release in gastric cancer patients. P=0.04, indicating publication bias; **(B)** Publication bias detection of exosomal lncRNA released by gastric cancer patients. The results showed that P＜0.01, indicating publication bias; **(C)** Publication bias detection of circRNA-related articles on exosomes released by gastric cancer patients. The results showed that P=0.46 greater than 0.05 indicated no publication bias.

**C**

**

**

**Fig. S2 Deek's funnel plot asymmetry test was used to detect articles publication bias. (A)** Detection of publication bias in articles related to exosomal miRNA release in gastric cancer patients. P=0.04, indicating publication bias; **(B)** Publication bias detection of exosomal lncRNA released by gastric cancer patients. The results showed that P＜0.01, indicating publication bias; **(C)** Publication bias detection of circRNA-related articles on exosomes released by gastric cancer patients. The results showed that P=0.46 greater than 0.05 indicated no publication bias.

**A**

**
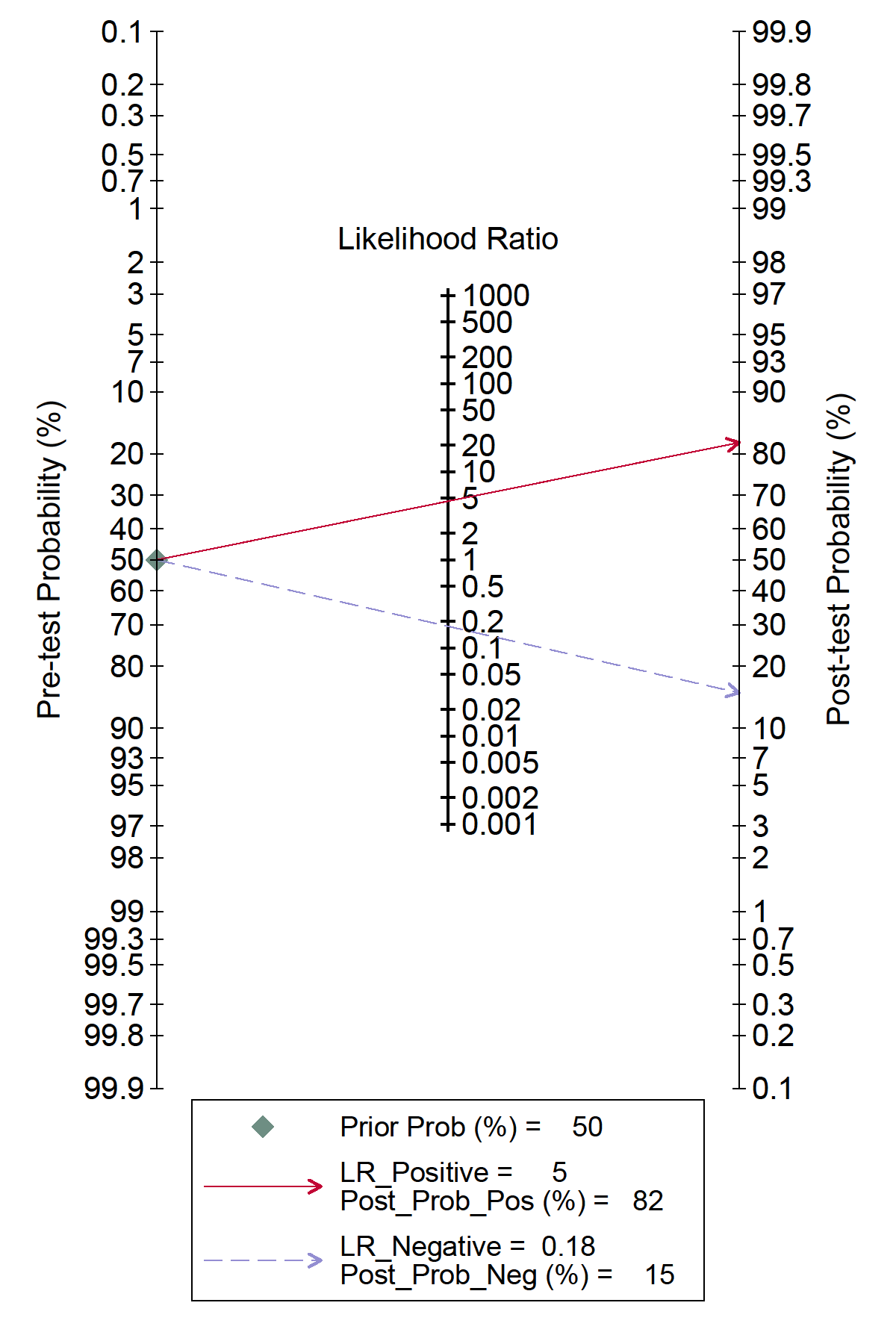
**

**Fig. S3 The clinical significance of exosomal non-coding RNA in the diagnosis of gastric cancer was evaluated by Fagan diagram. (A)** exosomal miRNA; **(B)** exosomal lncRNA; **(C)** exosomal circRNA.

**B**

**

**

**Fig. S3 The clinical significance of exosomal non-coding RNA in the diagnosis of gastric cancer was evaluated by Fagan diagram. (A)** exosomal miRNA; **(B)** exosomal lncRNA; **(C)** exosomal circRNA.

**C**

**

**

**Fig. S3 The clinical significance of exosomal non-coding RNA in the diagnosis of gastric cancer was evaluated by Fagan diagram. (A)** exosomal miRNA; **(B)** exosomal lncRNA; **(C)** exosomal circRNA.

**A**

**
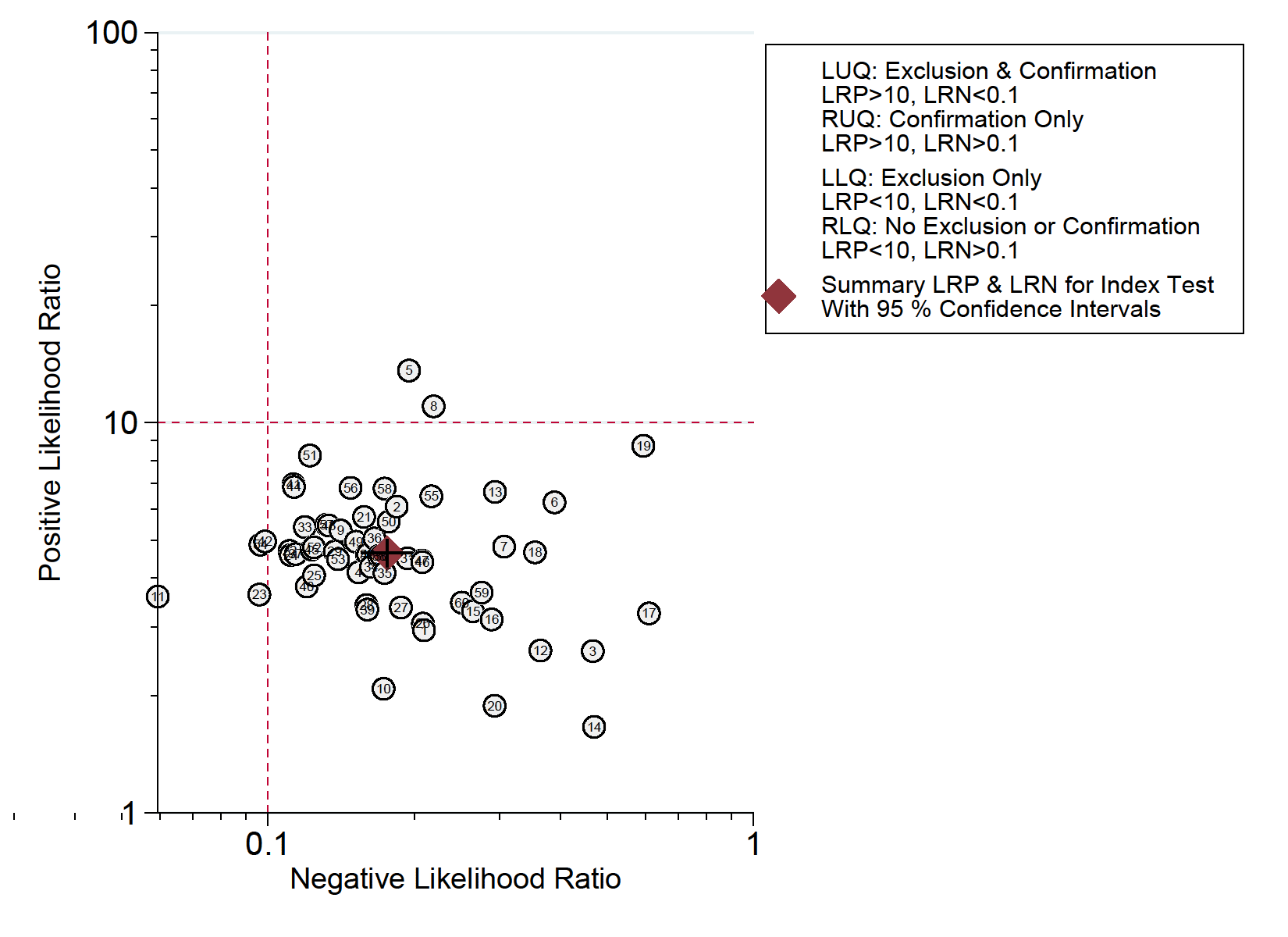
**

**Fig. S4 The likelihood ratio scattergram to analyze the clinical significance of exosomal non-coding RNA in the diagnosis of gastric cancer. (A)**exosomal miRNA; **(B)**exosomal lncRNA; **(C)**exosomal circRNA.

**B**

**

**

**Fig. S4 The likelihood ratio scattergram to analyze the clinical significance of exosomal non-coding RNA in the diagnosis of gastric cancer. (A)**exosomal miRNA; **(B)**exosomal lncRNA; **(C)**exosomal circRNA.

**C**

**

**

**Fig. S4 The likelihood ratio scattergram to analyze the clinical significance of exosomal non-coding RNA in the diagnosis of gastric cancer. (A)**exosomal miRNA; **(B)**exosomal lncRNA; **(C)**exosomal circRNA.

## Supplementary Table

**Table S1** Search Strategy

Search strategy in Web of Science (Aug 29, 2024)

| Search | Query | Results |
| --- | --- | --- |
| #1 | "((TS=(exocome)) OR TS=(exosomal)) OR TS=(extracellular vesicle) | 47930 |
| #2 | "(TS=(gastroesophag* OR stomach OR gastric OR gastrointestin*)) AND TS=(cancer OR neoplasm OR neoplasia OR carcinoma OR tumor) | 217793 |
| #3 | "TS=(Microrna or lncrna or long untranslated rna or long intergenic non-protein coding rna or circrnas or small interfering rna or small nuclear rna or piwi-interacting rna) | 205038 |
| #4 | "TS=(area under the curve or receiver operating characteristic or sensitivity or specificity or diagnosis) | 3506924 |
| #5 | "#1 AND #2 AND #3 AND #4 | 103 |

Search strategy in PubMed (Aug 29, 2024)

| Search | Query | Results |
| --- | --- | --- |
| #1 | exosomal OR exosome OR exosom* OR ( extracellular AND vesicle) | 58,106 |
| #2 | (gastroesophag* OR stomach OR gastric OR gastrointestin*) AND (cancer OR neoplasm OR neoplasia OR carcinoma OR tumor) | 333,919 |
| #3 | Microrna or lncrna or long untranslated rna or long intergenic non-protein coding rna or circrnas or small interfering rna or small nuclear rna or piwi-interacting rna | 327,073 |
| #4 | area under the curve or receiver operating characteristic or sensitivity or specificity or diagnosis | 15,642,237 |
| #5 | #1 AND #2 AND #3 AND #4 | 314 |

Search strategy in Science Direct (Aug 29, 2024)

| Search | Query | Results |
| --- | --- | --- |
| #1 | (exosomal OR exosome [Title Or abstract OR keywords]) AND (gastric cancer [Title Or abstract OR keywords]) AND (Microrna OR lncrna OR circrnas OR ncrna [Title Or abstract OR keywords]) | 38 |

Search strategy in Embase (Aug 29, 2024)

| Search | Query | Results |
| --- | --- | --- |
| #1 | exosome'/exp OR 'exosome' OR 'exosomal lncrna' OR 'exosomal' | 69,820 |
| #2 | (gastroesophag* OR stomach OR gastric OR gastrointestin*) AND (cancer OR neoplasm OR neoplasia OR carcinoma OR tumor) | 583,315 |
| #3 | micrornas' OR 'microrna' OR 'primary microrna' OR 'mirna' OR 'small temporal rna' OR 'rna, long noncoding' OR 'lncrna' OR 'long non-translated rna' OR 'long noncoding rna' OR 'long ncrnas' OR 'long untranslated rna' OR 'long intergenic non-protein coding rna' OR 'rna, circular' OR 'circrnas' OR 'closed circular rna' OR 'circular intronic rna' OR 'cirna' OR 'small interfering rna' OR 'small nuclear rna' OR 'small nucleolar rna' OR 'piwi-interacting rna' | 464,707 |
| #4 | sensitivity'/exp OR 'sensitivity' OR 'specificity'/exp OR 'specificity' OR 'sensitivity and specificity'/exp OR 'sensitivity and specificity' OR 'area under the curve'/exp OR 'area under the curve' OR 'auc'/exp OR 'auc' OR 'receiver operating characteristic'/exp OR 'receiver operating characteristic' OR 'roc' OR 'diagnosis' OR 'diagnoses' OR 'diagnoses and examinations' | 9,108,201 |
| #5 | #1 AND #2 AND #3 AND #4 | 573 |

Search strategy in Scopus (Aug 29, 2024)

| Search | Query | Results |
| --- | --- | --- |
| #1 | ALL ( exosomal OR exosome OR exosom* OR ( extracellular AND vesicle ) ) | 388,666 |
| #2 | ALL ( ( gastroesophag* OR stomach OR gastric OR gastrointestin* ) AND ( cancer OR neoplasm OR neoplasia OR carcinoma OR tumor ) ) | 1,759,115 |
| #3 | ALL ( microrna OR lncrna OR long AND untranslated AND rna OR long AND intergenic AND non-protein AND coding AND rna OR circrnas OR small AND interfering AND rna OR small AND nuclear AND rna OR piwi-interacting AND rna ) | 339 |
| #4 | #1 AND #2 AND #3 | 55 |

Search strategy in Medline (Aug 29, 2024)

| Search | Query | Results |
| --- | --- | --- |
| #1 | ((TS=(exocome)) OR TS=(exosomal)) OR TS=(extracellular vesicle) | 41343 |
| #2 | (TS=(gastroesophag* OR stomach OR gastric OR gastrointestin*)) AND TS=(cancer OR neoplasm OR neoplasia OR carcinoma OR tumor) | 282784 |
| #3 | TS=(Microrna or lncrna or long untranslated rna or long intergenic non-protein coding rna or circrnas or small interfering rna or small nuclear rna or piwi-interacting rna) | 266626 |
| #4 | TS=(area under the curve or receiver operating characteristic or sensitivity or specificity or diagnosis) | 6227079 |
| #5 | #1 AND #2 AND #3 AND #4 | 74 |

**Table S2** The information for exosomal ncRNAs.

| Author | Year | Types of sample | Exosome isolation method | TEM | NTA | Exosomr Diameter range | Exosomoe proteins |
| --- | --- | --- | --- | --- | --- | --- | --- |
| **MiRNA** |  |  |  |  |  |  |  |
| X. Zhou | 2015 | Plasma | ExoQuick Exosome Precipitation Solution | None | None | None | None |
| Yun. Zhang | 2021 | Serum | ExoQuick Exosome Precipitation Solution | None | None | None | CD9, and TSG-101 |
| D. Yu | 2024 | Serum | ultracentrifugation | Yes | Yes | 160nm | CD9, CD63, CD66b and Calnexin |
| J. Yang | 2021 | Plasma | miRNeasy Serum/Plasma Kit | Yes | Yes | None | CD63 and TSG101 |
| H. Yang | 2018 | Serum | ExoQuick-TC™ | Yes | Yes | 50 - 100 nm | CD81 |
| S. Wei | 2020 | Serum | exoEasy Maxi Kit | Yes | Yes | None | CD9, CD63 and TSG101 |
| Z. Wang | 2023 | Serum | exoEasyMaxi kit and ultracentrifugation | None | None | None | None |
| N. Wang | 2017 | Serum | mirVana PARIS Kit | Yes | None | 60 – 150 nm | CD63 |
| J. Wang | 2024 | Serum | Centrifugation | Yes | Yes | 30 – 150 nm | CD9 and CD63 |
| J. Wang | 2022 | Serum | exoRNeasy Serum Starter Kit | Yes | Yes | 30 – 120nm | CD9, CD63, TSG101 and calnexin |
| S. Tang | 2020 | Serum | exosome precipitation solution | None | None | None | None |
| Y. Shi | 2020 | Serum | RiboTM Exosome Isolation Reagent Kit | Yes | None | None | CD63 and TSG101 |
| Z. Ren | 2022 | Serum | Ultracentrifugation combined with filtration | Yes | None | None | CD63, TSG101 and calnexin |
| X. Lu | 2021 | Serum | differential ultracentrifugation | Yes | Yes | approximately 141 nm | CD9 and CD63 |
| H. Kahroba | 2022 | Serum | serial ultracentrifugation | Yes | None | 100 – 200 nm | CD9, CD81, CD63 and Calnexin |
| R. Ji | 2019 | Serum | ExoQuick | Yes | None | 40 – 100 nm | CD63 and CD9 |
| Z. Huang | 2017 | Serum | ExoQuick | None | None | None | None |
| J. He | 2023 | Plasma | ExoQuick exosome precipitation solutions | Yes | None | about 100nm | CD63, CD9 and TGS101 |
| L. Chang | 2021 | Serum | ExoQuick-TC™Exosome precipitation solution | Yes | Yes | 20 – 120 nm | CD63 and CD9 |
| L. Ge | 2020 | Serum | exoRNeasy Serum/Plasma Kits | Yes | Yes | 100 nm | CD9, TSG101 and Alix |
| A. Rincón-Riveros | 2023 | Plasma | Size Exclusion Chromatography | Yes | Yes | 150 nm | CD81 and CD63 |
| **LncRNA** |  |  |  |  |  |  |  |
| H. Zhou | 2020 | Serum | ExoQuick-TCTM Exosome Precipitation Solution | Yes | None | 50 – 150 nm | CD63 and TSG101 |
| P. Zheng | 2020 | Plasma | ExoQuick solution or ExoQuickTC solution | Yes | Yes | 80 – 120 nm | CD63, TSG101 and Tubulin |
| R. Zhao | 2018 | Serum | Centrifugation/None | None | None | None | None |
| Yan. Zhang | 2021 | Serum | Immunomagnetic bead, CD63-IMB | Yes | Yes | 30 – 150 nm | CD9, CD81 and Calreticulin |
| X. Zhang | 2018 | Serum | None | None | None | None | None |
| C. Zhang | 2023 | Plasma | Ultrafiltration | Yes | None | None | None |
| H. Xu | 2020 | Serum | ExoQuick Exosome Precipitation Solution | None | None | None | CD63, TSG101 and Flotillin-1 |
| K. Xiao | 2021 | Serum | exoEasyMaxi kit and ultracentrifugation | Yes | Yes | 125.6±51.7 and 119.5±51.8 nm, | CD9, CD63, TSG101 and Calnexin |
| B. Xia | 2023 | Serum | exosome extraction reagents | Yes | Yes | None | CD9, CD81, CD63, and Alix |
| H. Piao | 2020 | Plasma | Differential centrifugation | Yes | None | None | CD63, CD81 and HSP70 |
| L. Pan | 2017 | Serum | exosome quick extraction solution | None | None | None | CD9 and CD81 |
| L. Lin | 2018 | Plasma | ultracentrifugation and discontinuous iodixanol gradient methods | Yes | Yes | None | CD9, CD63 and Tubulin |
| S. Li | 2020 | Serum | exosome extraction kit | Yes | Yes | 60.1 nm | CD54, CD9, TSG101 and ARF6 |
| Q. Li | 2015 | Plasma | Total Exosome Isolation Reagent | Yes | None | 30 – 110 nm | None |
| X. Guo | 2023 | Plasma | exoRNeasy Serum/Plasma Kit | Yes | Yes | 50 – 200 nm | CD9, CD63, TSG101 and Calnexin |
| X. Guo | 2020 | Serum | ultracentrifugation | Yes | Yes | 80 – 120 nm | CD9, CD63 and Tubulin |
| C. Cai | 2019 | Serum | HiPure Exosome kits | Yes | Yes | 59.8 nm | CD54 and TSG101 |
| **CircRNA** |  |  |  |  |  |  |  |
| P. Zheng | 2022 | Plasma | ExoQuick solution | Yes | Yes | 80 – 120 nm | None |
| Z. Zhang | 2022 | Serum | Total Exosome Isolation Kits | Yes | None | no more than 200 nm | CD63, TSG101 and ALIX |
| X. Zang | 2024 | Plasma | ExoQuick Plasma kit | Yes | Yes | None | CD9, CD81 and CD63 |
| J. You | 2023 | Serum | Total Exosome Isolation Kit | Yes | None | ~ 100 nm | None |
| X. Yang | 2023 | Plasma | ExoQuick solution Kit | Yes | None | None | None |
| K. Xiao | 2022 | Serum | exoEasyMaxi kit and ultracentrifugation | None | None | None | None |
| Y. Wang | 2021 | Serum | exosome quick extraction solution | Yes | Yes | 100 nm | None |
| X. Tao | 2020 | Plasma | exosome isolation reagent | None | None | None | None |
| W. Tang | 2018 | Plasma | Hieff™ Quick exosome isolation kit | None | None | None | None |
| Y. Shao | 2020 | Plasma | total exosome isolation reagent | None | None | None | None |
| X. Li | 2023 | Plasma | size-exclusion chromatography and ExoQuickTM Exosome Precipitation Solution | Yes | Yes | 40 – 200 nm | CD9, CD81, TSG101 and Calnexin |
| R. Li | 2023 | Plasma | ExoQuick exosome precipitation solution | None | None | None | None |
| X. Huang | 2023 | Serum | ExoQuick exosome precipitation solution | Yes | Yes | None | CD63, CD81, TSG101 and Calnexin |
| X. Sun | 2022 | Plasma | None | None | None | None | None |

**Table S3** Values the diagnostic effectiveness of the included studies

| Author | RNA | TP | FP | FN | TN | Sen | Spe | AUC | DLR+ | DLR- | DOR |
| --- | --- | --- | --- | --- | --- | --- | --- | --- | --- | --- | --- |
| **miRNA** |  |  |  |  |  |  |  |  |  |  |  |
| D. Yu(1) | miR-223-3p | 23 | 8 | 38 | 41 | 0.38 | 0.84 | 0.59 | 2.38 | 0.74 | 3.22 |
| D. Yu(2) | miR-223-3p | 26 | 10 | 35 | 26 | 0.42 | 0.73 | 0.56 | 1.56 | 0.79 | 1.96 |
| D. Yu(3) | miR-223-3p | 13 | 58 | 3 | 27 | 0.83 | 0.32 | 0.55 | 1.22 | 0.53 | 2.30 |
| D. Yu(4) | miR-223-3p | 18 | 19 | 27 | 66 | 0.41 | 0.78 | 0.59 | 1.86 | 0.76 | 2.46 |
| D. Yu(5) | miR425-5p | 29 | 4 | 32 | 45 | 0.47 | 0.91 | 0.71 | 5.22 | 0.58 | 8.97 |
| D. Yu(6) | miR425-5p | 50 | 18 | 11 | 18 | 0.82 | 0.49 | 0.69 | 1.61 | 0.37 | 4.38 |
| D. Yu(7) | miR425-5p | 15 | 45 | 1 | 40 | 0.94 | 0.47 | 0.71 | 1.77 | 0.13 | 13.89 |
| D. Yu(8) | miR425-5p | 22 | 9 | 23 | 76 | 0.49 | 0.89 | 0.70 | 4.45 | 0.57 | 7.77 |
| D. Yu(9) | miR-223-3p+miR425-5p | 41 | 13 | 20 | 36 | 0.68 | 0.73 | 0.71 | 2.52 | 0.44 | 5.74 |
| D. Yu(10) | miR-223-3p+miR425-5p | 34 | 9 | 27 | 27 | 0.55 | 0.76 | 0.70 | 2.29 | 0.59 | 3.87 |
| D. Yu(11) | miR-223-3p+miR425-5p | 15 | 45 | 1 | 40 | 0.94 | 0.47 | 0.70 | 1.77 | 0.13 | 13.89 |
| D. Yu(12) | miR-223-3p+miR425-5p | 22 | 10 | 23 | 75 | 0.49 | 0.88 | 0.69 | 4.08 | 0.58 | 7.05 |
| H. Kahroba(1) | Exo-miR-10a-5p | 33 | 10 | 10 | 30 | 0.76 | 0.74 | 0.80 | 2.92 | 0.32 | 9.01 |
| H. Kahroba(2) | Exo-miR-18a-5p | 31 | 12 | 12 | 28 | 0.72 | 0.71 | 0.72 | 2.48 | 0.39 | 6.30 |
| H. Kahroba(3) | Exo-miR-19b-3p | 32 | 12 | 11 | 28 | 0.74 | 0.69 | 0.78 | 2.39 | 0.38 | 6.34 |
| H. Kahroba(4) | Exo-miR-215-5p | 29 | 13 | 14 | 27 | 0.68 | 0.67 | 0.74 | 2.06 | 0.48 | 4.31 |
| H. Kahroba(5) | Exo-miR-10a-5p+miR-18a-5p+miR-19b-3p+miR-215-5p | 31 | 11 | 12 | 29 | 0.73 | 0.72 | 0.81 | 2.61 | 0.38 | 6.95 |
| H. Yang | miR-423-5p | 65 | 34 | 15 | 46 | 0.81 | 0.58 | 0.76 | 1.93 | 0.33 | 5.89 |
| J. He(1) | miR-31 | 44 | 18 | 16 | 45 | 0.73 | 0.72 | 0.73 | 2.61 | 0.38 | 6.95 |
| J. He(2) | miR-192 | 47 | 11 | 13 | 52 | 0.78 | 0.83 | 0.81 | 4.59 | 0.27 | 17.31 |
| J. He(3) | miR-375 | 35 | 5 | 25 | 58 | 0.59 | 0.92 | 0.75 | 7.38 | 0.45 | 16.55 |
| J. He(4) | miR-31+miR-192 +miR-375 | 49 | 13 | 11 | 50 | 0.82 | 0.80 | 0.84 | 4.10 | 0.23 | 18.22 |
| J. Wang(1) | miR-21-5p | 18 | 0 | 15 | 33 | 0.54 | 1.00 | 0.76 | 37.01 | 0.46 | 80.46 |
| J. Wang(2) | miR-26a-5p | 23 | 0 | 10 | 33 | 0.69 | 1.00 | 0.74 | 47.02 | 0.31 | 151.68 |
| J. Wang(3) | miR-27a-3p | 18 | 4 | 15 | 29 | 0.56 | 0.89 | 0.51 | 5.09 | 0.49 | 10.30 |
| J. Wang(1) | miR–10401–3p | 22 | 7 | 2 | 17 | 0.92 | 0.71 | 0.83 | 3.17 | 0.11 | 28.15 |
| J. Wang(2) | miR–1255–5p | 20 | 5 | 4 | 19 | 0.83 | 0.79 | 0.83 | 3.95 | 0.22 | 18.37 |
| J. Wang(3) | miR–6736–5p | 23 | 9 | 1 | 15 | 0.96 | 0.63 | 0.81 | 2.59 | 0.06 | 40.86 |
| J. Yang(1) | miR-195-5p | 15 | 5 | 5 | 15 | 0.75 | 0.75 | 0.75 | 3.00 | 0.33 | 9.00 |
| J. Yang(2) | miR-195-5p | 60 | 23 | 28 | 65 | 0.68 | 0.74 | 0.76 | 2.62 | 0.43 | 6.05 |
| J. Yang(3) | miR-211-5p | 16 | 5 | 4 | 15 | 0.79 | 0.73 | 0.80 | 2.93 | 0.29 | 10.17 |
| J. Yang(4) | miR-211-5p | 53 | 8 | 35 | 80 | 0.60 | 0.91 | 0.80 | 6.67 | 0.44 | 15.17 |
| J. Yang(5) | miR-195-5p+miR-211-5p | 17 | 5 | 3 | 15 | 0.84 | 0.73 | 0.83 | 3.11 | 0.22 | 14.19 |
| J. Yang(6) | miR-195-5p+miR-211-5p | 60 | 10 | 28 | 78 | 0.68 | 0.89 | 0.82 | 6.18 | 0.36 | 17.19 |
| L. Chang | miR-1228 | 36 | 4 | 10 | 26 | 0.78 | 0.87 | 0.87 | 6.00 | 0.25 | 23.72 |
| L. Ge(1) | miR-1307-3p,piR-019308,piR-004918,piR-018569 | 57 | 14 | 13 | 46 | 0.81 | 0.77 | 0.85 | 3.52 | 0.25 | 14.27 |
| L. Ge(2) | miR-1307-3p,piR-019308,piR-004918,piR-018569 | 40 | 5 | 30 | 55 | 0.57 | 0.92 | 0.82 | 7.13 | 0.47 | 15.24 |
| L. Ge(3) | miR-1307-3p,piR-019308,piR-004918,piR-018569 | 30 | 3 | 40 | 57 | 0.43 | 0.95 | 0.75 | 8.60 | 0.60 | 14.33 |
| L. Ge(4) | miR-1307-3p,piR-019308,piR-004918,piR-018569 | 31 | 2 | 39 | 58 | 0.44 | 0.97 | 0.73 | 14.67 | 0.58 | 25.41 |
| N. Wang(1) | miR-106a-5p | 17 | 6 | 3 | 14 | 0.84 | 0.72 | 0.81 | 3.00 | 0.22 | 13.50 |
| N. Wang(2) | miR-106a-5p | 19 | 8 | 1 | 12 | 0.95 | 0.60 | 0.81 | 2.38 | 0.08 | 28.51 |
| N. Wang(3) | miR-19b-3p | 17 | 4 | 3 | 16 | 0.84 | 0.78 | 0.83 | 3.82 | 0.21 | 18.62 |
| N. Wang(4) | miR-19b-3p | 57 | 9 | 33 | 81 | 0.63 | 0.90 | 0.79 | 6.30 | 0.41 | 15.32 |
| N. Wang(5) | miR-30a-5p+miR-17-5p | 53 | 16 | 37 | 74 | 0.59 | 0.82 | 0.77 | 3.28 | 0.50 | 6.56 |
| N. Wang(6) | miR-30a-5p+miR-17-5p | 65 | 14 | 25 | 76 | 0.72 | 0.84 | 0.81 | 4.50 | 0.33 | 13.50 |
| R. Ji | miR-374a-5p | 45 | 1 | 14 | 33 | 0.76 | 0.97 | 0.92 | 25.33 | 0.25 | 102.40 |
| S. Tang(1) | miR-9-5p | 29 | 1 | 7 | 11 | 0.80 | 0.91 | 0.92 | 8.89 | 0.22 | 40.44 |
| S. Tang(2) | let-7g-5p | 27 | 1 | 9 | 11 | 0.74 | 0.91 | 0.85 | 8.22 | 0.29 | 28.78 |
| S. Tang(3) | let-7c-5p | 29 | 1 | 7 | 11 | 0.80 | 0.91 | 0.89 | 8.89 | 0.22 | 40.44 |
| S. Tang(4) | miR-146b-5p | 26 | 2 | 10 | 10 | 0.71 | 0.82 | 0.78 | 3.94 | 0.35 | 11.15 |
| S. Tang(5) | miR-92b-3p | 24 | 1 | 12 | 11 | 0.67 | 0.91 | 0.79 | 7.44 | 0.36 | 20.53 |
| S. Tang(6) | miR-101-3p | 27 | 1 | 9 | 11 | 0.75 | 0.91 | 0.81 | 8.33 | 0.27 | 30.34 |
| S. Tang(7) | miR-21-5p | 36 | 1 | 0 | 11 | 1.00 | 0.91 | 1.00 | 8.55 | 0.02 | 558.82 |
| S. Tang(8) | miR-26a-5p | 31 | 1 | 5 | 11 | 0.86 | 0.91 | 0.96 | 9.56 | 0.15 | 62.13 |
| S. Tang(9) | miR-92b-3p | 29 | 10 | 21 | 40 | 0.58 | 0.80 | 0.71 | 2.90 | 0.53 | 5.52 |
| S. Tang(10) | let-7g-5p | 27 | 6 | 23 | 44 | 0.54 | 0.88 | 0.76 | 4.50 | 0.52 | 8.61 |
| S. Tang(11) | miR-146b-5p | 23 | 9 | 27 | 41 | 0.46 | 0.82 | 0.67 | 2.56 | 0.66 | 3.88 |
| S. Tang(12) | miR-9-5p | 25 | 8 | 25 | 42 | 0.50 | 0.84 | 0.63 | 3.13 | 0.60 | 5.25 |
| S. Tang(13) | miR-92b-3p+let-7g-5p | 32 | 11 | 18 | 39 | 0.64 | 0.78 | 0.78 | 2.91 | 0.46 | 6.30 |
| S. Tang(14) | miR-92b-3p+miR-146b-5p | 30 | 9 | 20 | 41 | 0.60 | 0.82 | 0.74 | 3.33 | 0.49 | 6.83 |
| S. Tang(15) | miR-146b-5p+miR-9-5p | 22 | 6 | 28 | 44 | 0.44 | 0.88 | 0.71 | 3.67 | 0.64 | 5.76 |
| S. Tang(16) | miR-92b-3p+let-7g-5p+miR-146b-5p | 29 | 7 | 21 | 43 | 0.58 | 0.86 | 0.77 | 4.14 | 0.49 | 8.48 |
| S. Tang(17) | miR-92b-3p+let-7g-5p+miR-9-5p | 30 | 9 | 20 | 41 | 0.60 | 0.82 | 0.77 | 3.33 | 0.49 | 6.83 |
| S. Tang(18) | miR-92b-3p+miR-146b-5p+miR-9-5p | 34 | 13 | 16 | 37 | 0.68 | 0.74 | 0.75 | 2.62 | 0.43 | 6.05 |
| S. Tang(19) | miR-92b-3p+let-7g-5p+miR-146b-5p+miR-9-5p | 30 | 8 | 20 | 42 | 0.60 | 0.84 | 0.77 | 3.75 | 0.48 | 7.87 |
| S. Wei | miR-15b-3p | 87 | 28 | 21 | 80 | 0.81 | 0.74 | 0.82 | 3.12 | 0.26 | 12.13 |
| X. Lu | miR-92a-3p | 86 | 15 | 45 | 107 | 0.66 | 0.88 | 0.83 | 5.50 | 0.39 | 14.23 |
| X. Zhou(1) | miR-185+miR-20a+miR-210+miR-25+miR-92b | 66 | 18 | 35 | 73 | 0.65 | 0.80 | 0.77 | 3.25 | 0.44 | 7.43 |
| X. Zhou(2) | miR-185+miR-20a+miR-210+miR-25+miR-92b | 27 | 8 | 3 | 22 | 0.89 | 0.73 | 0.86 | 3.30 | 0.15 | 21.87 |
| X. Zhou(3) | miR-185+miR-20a+miR-210+miR-25+miR-92b | 44 | 13 | 27 | 48 | 0.62 | 0.78 | 0.74 | 2.82 | 0.49 | 5.78 |
| X. Zhou(4) | miR-185+miR-20a+miR-210+miR-25+miR-92b | 26 | 3 | 6 | 15 | 0.82 | 0.86 | 0.87 | 5.86 | 0.21 | 27.98 |
| Y. Shi(1) | miR-1246 | 111 | 16 | 6 | 66 | 0.95 | 0.80 | 0.95 | 4.75 | 0.06 | 76.00 |
| Y. Shi(2) | miR-1246 | 70 | 7 | 15 | 43 | 0.82 | 0.86 | 0.91 | 5.86 | 0.21 | 27.98 |
| Y. Shi(3) | miR-1246 | 24 | 50 | 4 | 0 | 0.86 | 0.74 | 0.84 | 0.86 | 15.84 | 0.05 |
| Y. Shi(4) | miR-1246 | 22 | 6 | 6 | 24 | 0.79 | 0.80 | 0.81 | 3.95 | 0.26 | 15.05 |
| Yun. Zhang(1) | miR-215-5p | 81 | 2 | 37 | 68 | 0.69 | 0.97 | 0.87 | 23.00 | 0.32 | 71.96 |
| Yun. Zhang(2) | miR-215-5p | 77 | 3 | 41 | 57 | 0.65 | 0.95 | 0.81 | 13.00 | 0.37 | 35.29 |
| Yun. Zhang(3) | miR-215-5p | 27 | 15 | 2 | 74 | 0.93 | 0.83 | 0.91 | 5.47 | 0.08 | 64.89 |
| Z. Huang(1) | miR10b-5p+miR132-3p+miR185-5p+miR195-5p+miR20a3p+miR296-5p | 130 | 55 | 73 | 112 | 0.64 | 0.67 | 0.70 | 1.94 | 0.54 | 3.61 |
| Z. Huang(2) | miR10b-5p+miR132-3p+miR185-5p+miR195-5p+miR20a3p+miR296-5p | 46 | 23 | 3 | 24 | 0.94 | 0.51 | 0.76 | 1.92 | 0.12 | 16.31 |
| Z. Huang(3) | miR10b-5p+miR132-3p+miR185-5p+miR195-5p+miR20a3p+miR296-5p | 105 | 44 | 49 | 76 | 0.68 | 0.63 | 0.70 | 1.84 | 0.51 | 3.62 |
| Z. Ren(1) | hsa-miR-1273g-3p | 20 | 0 | 16 | 20 | 0.55 | 1.00 | 0.78 | 41.00 | 0.81 | 50.93 |
| Z. Ren(2) | hsa-miR-619-5p | 20 | 1 | 16 | 19 | 0.55 | 0.94 | 0.75 | 9.17 | 0.48 | 19.15 |
| Z. Ren(3) | hsa-miR-4793-3p | 31 | 7 | 5 | 13 | 0.85 | 0.64 | 0.78 | 2.36 | 0.23 | 10.07 |
| Z. Wang(1) | NSD1 | 90 | 14 | 20 | 39 | 0.82 | 0.74 | 0.85 | 3.15 | 0.24 | 12.97 |
| Z. Wang(2) | FBXO7 | 57 | 10 | 53 | 43 | 0.52 | 0.82 | 0.69 | 2.89 | 0.59 | 4.93 |
| Z. Wang(3) | NSD1+FBXO7 | 86 | 4 | 24 | 49 | 0.78 | 0.92 | 0.85 | 9.75 | 0.24 | 40.78 |
| A. Rincón-Riveros | hsa-miR-451a+hsa-miR-126-3p+hsamiR-92a-3p | 20 | 2 | 0 | 8 | 1.00 | 0.79 | 0.92 | 8.20 | 0.06 | 139.46 |
| **LncRNA** |  |  |  |  |  |  |  |  |  |  |  |
| B. Xia | LINC00691 | 80 | 11 | 14 | 27 | 0.85 | 0.71 | 0.86 | 2.93 | 0.21 | 13.87 |
| C. Cai | Lnc RNA psck2-2:1 | 53 | 4 | 10 | 25 | 0.84 | 0.87 | 0.90 | 6.46 | 0.18 | 35.14 |
| C. Zhang | CCAT1 | 13 | 5 | 7 | 15 | 0.65 | 0.75 | 0.72 | 2.60 | 0.47 | 5.57 |
| H. Piao | CEBPA-AS1 | 247 | 17 | 34 | 63 | 0.88 | 0.79 | 0.82 | 4.19 | 0.15 | 27.59 |
| H. Xu(1) | MIAT | 89 | 3 | 20 | 47 | 0.82 | 0.94 | 0.89 | 13.67 | 0.19 | 71.37 |
| H. Xu(2) | MIAT | 71 | 5 | 38 | 43 | 0.65 | 0.90 | 0.79 | 6.50 | 0.39 | 16.71 |
| H. Zhou | lncRNA H19 | 60 | 12 | 21 | 66 | 0.74 | 0.84 | 0.85 | 4.63 | 0.31 | 14.94 |
| K. Xiao | lncRNA CCAT1 | 90 | 6 | 23 | 77 | 0.80 | 0.93 | 0.89 | 11.43 | 0.22 | 53.13 |
| L. Lin(1) | lncUEGC1 | 45 | 10 | 6 | 50 | 0.88 | 0.83 | 0.88 | 5.18 | 0.14 | 35.80 |
| L. Lin(2) | lncUEGC2 | 46 | 26 | 5 | 34 | 0.90 | 0.57 | 0.76 | 2.09 | 0.18 | 11.93 |
| L. Lin(3) | lncUEGC1 | 22 | 16 | 1 | 44 | 0.96 | 0.73 | 0.85 | 3.56 | 0.05 | 64.88 |
| L. Lin(4) | lncUEGC2 | 17 | 17 | 6 | 43 | 0.74 | 0.72 | 0.75 | 2.64 | 0.36 | 7.32 |
| L. Lin(5) | lncUEGC1 | 17 | 2 | 6 | 16 | 0.74 | 0.89 | 0.84 | 6.73 | 0.29 | 23.03 |
| L. Lin(6) | lncUEGC2 | 17 | 8 | 6 | 10 | 0.74 | 0.56 | 0.65 | 1.68 | 0.46 | 3.62 |
| L. Pan | ZFAS1 | 32 | 9 | 8 | 28 | 0.80 | 0.76 | 0.84 | 3.33 | 0.26 | 12.66 |
| P. Zheng | lnc-SLC2A12-10:1 | 47 | 15 | 13 | 45 | 0.78 | 0.75 | 0.78 | 3.12 | 0.29 | 10.64 |
| Q. Li | LINC00152 | 38 | 12 | 41 | 69 | 0.48 | 0.85 | 0.66 | 3.20 | 0.61 | 5.23 |
| R. Zhao(1) | HOTTIP | 88 | 18 | 38 | 102 | 0.70 | 0.85 | 0.83 | 4.67 | 0.35 | 13.22 |
| R. Zhao(2) | HOTTIP | 55 | 6 | 71 | 114 | 0.44 | 0.95 | 0.83 | 8.80 | 0.59 | 14.93 |
| S. Li | lnc-GNAQ-6:1 | 36 | 12 | 7 | 15 | 0.84 | 0.56 | 0.74 | 1.91 | 0.29 | 6.68 |
| X. Guo(1) | lncRNA GClnc1 | 97 | 24 | 15 | 135 | 0.87 | 0.85 | 0.94 | 5.80 | 0.15 | 37.93 |
| X. Guo(2) | lncRNA GClnc1 | 102 | 13 | 10 | 54 | 0.91 | 0.80 | 0.93 | 4.55 | 0.11 | 40.44 |
| X. Guo(3) | lncRNA GClnc1 | 104 | 22 | 8 | 64 | 0.93 | 0.74 | 0.91 | 3.58 | 0.09 | 37.81 |
| X. Guo(4) | lncRNA GClnc1 | 102 | 62 | 10 | 250 | 0.91 | 0.80 | 0.93 | 4.55 | 0.11 | 40.44 |
| X. Guo(5) | lncRNA GClnc1 | 84 | 24 | 9 | 84 | 0.90 | 0.78 | 0.88 | 4.09 | 0.13 | 31.91 |
| X. Guo(6) | lncRNA GClnc1 | 79 | 15 | 14 | 39 | 0.85 | 0.73 | 0.85 | 3.15 | 0.21 | 15.32 |
| X. Guo(7) | lncRNA GClnc1 | 80 | 11 | 13 | 32 | 0.86 | 0.74 | 0.84 | 3.31 | 0.19 | 17.48 |
| X. Guo(8) | lncRNA GClnc1 | 82 | 53 | 11 | 152 | 0.88 | 0.74 | 0.87 | 3.38 | 0.16 | 20.87 |
| X. Guo(9) | lncRNA GClnc1 | 96 | 27 | 12 | 115 | 0.89 | 0.81 | 0.90 | 4.68 | 0.14 | 34.49 |
| X. Guo(10) | lncRNA GClnc1 | 93 | 7 | 15 | 30 | 0.86 | 0.80 | 0.87 | 4.30 | 0.18 | 24.57 |
| X. Guo(11) | lncRNA GClnc1 | 91 | 12 | 17 | 52 | 0.84 | 0.81 | 0.85 | 4.42 | 0.20 | 22.39 |
| X. Guo(12) | lncRNA GClnc1 | 94 | 46 | 14 | 197 | 0.87 | 0.81 | 0.88 | 4.58 | 0.16 | 28.53 |
| X. Guo(13) | lncRNA GClnc1 | 118 | 18 | 13 | 90 | 0.90 | 0.83 | 0.90 | 5.29 | 0.12 | 43.93 |
| X. Guo(14) | lncRNA GClnc1 | 114 | 11 | 17 | 43 | 0.87 | 0.79 | 0.89 | 4.14 | 0.16 | 25.17 |
| X. Guo(15) | lncRNA GClnc1 | 113 | 9 | 18 | 34 | 0.86 | 0.79 | 0.86 | 4.10 | 0.18 | 23.11 |
| X. Guo(16) | lncRNA GClnc1 | 113 | 35 | 18 | 170 | 0.86 | 0.83 | 0.89 | 5.06 | 0.17 | 29.99 |
| X. Guo(17) | lncRNA GClnc1 | 139 | 28 | 14 | 114 | 0.91 | 0.80 | 0.91 | 4.55 | 0.11 | 40.44 |
| X. Guo(18) | lncRNA GClnc1 | 132 | 7 | 21 | 30 | 0.86 | 0.80 | 0.89 | 4.30 | 0.18 | 24.57 |
| X. Guo(19) | lncRNA GClnc1 | 135 | 17 | 18 | 47 | 0.88 | 0.74 | 0.87 | 3.38 | 0.16 | 20.87 |
| X. Guo(20) | lncRNA GClnc1 | 139 | 58 | 14 | 185 | 0.91 | 0.76 | 0.90 | 3.79 | 0.12 | 32.02 |
| X. Guo(21) | lncRNA GClnc1 | 202 | 14 | 22 | 94 | 0.90 | 0.87 | 0.94 | 6.92 | 0.11 | 60.25 |
| X. Guo(22) | lncRNA GClnc1 | 206 | 10 | 18 | 44 | 0.92 | 0.81 | 0.92 | 4.84 | 0.10 | 49.01 |
| X. Guo(23) | lncRNA GClnc1 | 199 | 7 | 25 | 36 | 0.89 | 0.83 | 0.92 | 5.24 | 0.13 | 39.51 |
| X. Guo(24) | lncRNA GClnc1 | 202 | 27 | 22 | 178 | 0.90 | 0.87 | 0.94 | 6.92 | 0.11 | 60.25 |
| X. Guo(25) | lncRNA GClnc1 | 235 | 27 | 26 | 115 | 0.90 | 0.81 | 0.92 | 4.74 | 0.12 | 38.35 |
| X. Guo(26) | lncRNA GClnc1 | 217 | 7 | 44 | 30 | 0.83 | 0.81 | 0.90 | 4.37 | 0.21 | 20.81 |
| X. Guo(27) | lncRNA GClnc1 | 217 | 12 | 44 | 52 | 0.83 | 0.81 | 0.89 | 4.37 | 0.21 | 20.81 |
| X. Guo(28) | lncRNA GClnc1 | 227 | 46 | 34 | 197 | 0.87 | 0.81 | 0.91 | 4.58 | 0.16 | 28.53 |
| X. Guo(1) | lncRNA-GC1 | 84 | 12 | 12 | 56 | 0.88 | 0.82 | 0.89 | 4.89 | 0.15 | 33.42 |
| X. Guo(2) | lncRNA-GC1 | 328 | 23 | 58 | 128 | 0.85 | 0.85 | 0.90 | 5.67 | 0.18 | 32.11 |
| X. Guo(3) | lncRNA-GC1 | 344 | 4 | 42 | 33 | 0.89 | 0.88 | 0.85 | 7.42 | 0.13 | 59.33 |
| X. Guo(4) | lncRNA-GC1 | 347 | 9 | 39 | 39 | 0.90 | 0.81 | 0.86 | 4.74 | 0.12 | 38.35 |
| X. Guo(5) | lncRNA-GC1 | 159 | 30 | 20 | 121 | 0.89 | 0.80 | 0.86 | 4.45 | 0.14 | 32.36 |
| X. Guo(6) | lncRNA-GC1 | 165 | 7 | 14 | 30 | 0.92 | 0.82 | 0.88 | 5.11 | 0.10 | 52.37 |
| X. Guo(7) | lncRNA-GC1 | 145 | 6 | 34 | 42 | 0.81 | 0.88 | 0.89 | 6.75 | 0.22 | 31.26 |
| X. Guo(8) | lncRNA-GC1 | 189 | 28 | 28 | 191 | 0.87 | 0.87 | 0.89 | 6.69 | 0.15 | 44.79 |
| X. Guo(9) | lncRNA-GC1 | 193 | 6 | 24 | 31 | 0.89 | 0.83 | 0.87 | 5.24 | 0.13 | 39.51 |
| X. Guo(10) | lncRNA-GC1 | 184 | 6 | 33 | 42 | 0.85 | 0.87 | 0.88 | 6.54 | 0.17 | 37.93 |
| X. Zhang | UFC1 | 47 | 16 | 13 | 59 | 0.78 | 0.79 | 0.86 | 3.71 | 0.28 | 13.34 |
| Y. Zhang | FRLnc1 | 42 | 7 | 10 | 23 | 0.81 | 0.77 | 0.86 | 3.52 | 0.25 | 14.27 |
| **CircRNA** |  |  |  |  |  |  |  |  |  |  |  |
| J. You | circ_0001789 | 39 | 0 | 31 | 50 | 0.55 | 1.00 | 0.82 | 79.00 | 0.62 | 126.60 |
| K. Xiao(1) | Chr10q11 | 86 | 15 | 26 | 29 | 0.77 | 0.66 | 0.73 | 2.26 | 0.35 | 6.50 |
| K. Xiao(2) | Chr1p11 | 92 | 10 | 20 | 34 | 0.82 | 0.77 | 0.82 | 3.57 | 0.23 | 15.25 |
| K. Xiao(3) | Chr7q11 | 90 | 18 | 22 | 26 | 0.80 | 0.59 | 0.75 | 1.95 | 0.34 | 5.76 |
| K. Xiao(4) | Chr10q11+Chr1p11+Chr7q11 | 82 | 7 | 30 | 37 | 0.73 | 0.84 | 0.84 | 4.56 | 0.32 | 14.20 |
| P. Zheng | hsa_circ_0015286 | 49 | 20 | 11 | 40 | 0.82 | 0.66 | 0.78 | 2.41 | 0.27 | 8.84 |
| R. Li | CircRNA CDR1as | 14 | 3 | 54 | 65 | 0.21 | 0.96 | 0.54 | 5.25 | 0.82 | 6.38 |
| W. Tang | circ-KIAA1244 | 48 | 8 | 14 | 17 | 0.77 | 0.68 | 0.75 | 2.41 | 0.34 | 7.12 |
| X. Huang | Hsa_circ_000200 | 49 | 28 | 1 | 22 | 0.98 | 0.44 | 0.71 | 1.75 | 0.05 | 38.46 |
| X. Li(1) | hsa_circ_0079439 | 163 | 3 | 77 | 60 | 0.68 | 0.95 | 0.86 | 13.60 | 0.34 | 40.38 |
| X. Li(2) | hsa_circ_0079439 | 112 | 3 | 65 | 60 | 0.63 | 0.95 | 0.84 | 12.60 | 0.39 | 32.35 |
| X. Li(3) | hsa_circ_0079439 | 51 | 3 | 12 | 60 | 0.81 | 0.96 | 0.91 | 20.25 | 0.20 | 102.32 |
| X. Tao | Hsa_circ_0000419 | 30 | 5 | 14 | 38 | 0.68 | 0.88 | 0.84 | 5.67 | 0.36 | 15.58 |
| X. Yang | circLPAR1 | 48 | 13 | 16 | 47 | 0.75 | 0.78 | 0.84 | 3.41 | 0.32 | 10.64 |
| X. Zang | circ50547 | 39 | 0 | 18 | 50 | 0.68 | 1.00 | 0.90 | 79.00 | 0.37 | 215.85 |
| Y. Shao | hsa_circ_0065149 | 19 | 4 | 20 | 37 | 0.49 | 0.90 | 0.64 | 4.90 | 0.57 | 8.65 |
| Y. Wang | CircITCH | 14 | 3 | 19 | 30 | 0.42 | 0.91 | 0.65 | 4.67 | 0.64 | 7.32 |
| Z. Zhang | circFCHO2 | 23 | 2 | 7 | 28 | 0.75 | 0.94 | 0.84 | 12.50 | 0.27 | 46.99 |
| X. Sun(1) | Hsa_circ_0002874 | 62 | 14 | 9 | 46 | 0.87 | 0.77 | 0.84 | 3.78 | 0.17 | 22.41 |
| X. Sun(2) | Hsa_circ_0002874 | 37 | 1 | 34 | 58 | 0.52 | 0.98 | 0.79 | 26.00 | 0.49 | 53.08 |

**Table S4** Diagnostic Performance of Frequently Studied Exosomal ncRNAs in Gastric Cancer

| **ncRNA Type** | **Candidate** | **AUC Range** | **Sensitivity Range** | | **Specificity Range** | **DOR Range** | | **Studies (n)** | **Consistency** |
| --- | --- | --- | --- | --- | --- | --- | --- | --- | --- |
| **miRNA** | miR-374a-5p^[26]^ | 0.92 | | 0.76–0.95 | 0.97 | | 102.4 | 1 | Single-study; needs validation |
|  | miR-1246^[11]^ | 0.84–0.95 | | 0.79–0.95 | 0.74–0.86 | | 15.05–76 | 4 | High sensitivity, moderate specificity |
|  | miR-21-5p^[20, 27]^ | 0.76–1.0 | | 0.54–1.0 | 0.91–1.0 | | 5.89–558.82 | 2 | Extreme variability (potential overfitting) |
|  | miR-19b-3p^[17, 25]^ | 0.78–0.83 | | 0.74–0.84 | 0.69–0.78 | | 6.34–18.62 | 2 | Moderately stable |
| **lncRNA** | lncRNA GClnc1^[48]^ | 0.84–0.94 | | 0.83–0.93 | 0.74–0.87 | | 15.32–60.25 | 28 | Most stable; multi-study validated |
|  | lncRNA-GC1^[13]^ | 0.85–0.90 | | 0.85–0.92 | 0.80–0.88 | | 32.11–59.33 | 10 | High reproducibility |
|  | CCAT1^[38, 41]^ | 0.72–0.89 | | 0.65–0.80 | 0.75–0.93 | | 5.57–53.13 | 2 | Outstanding specificity |
| **circRNA** | hsa_circ_0079439^[58]^ | 0.84–0.91 | | 0.63–0.81 | 0.95–0.96 | | 32.35–102.32 | 3 | Exceptionally high specificity |
|  | circLPAR1^[60]^ | 0.84 | | 0.75 | 0.78 | | 10.64 | 1 | Single-study; requires validation |
